# A simple method for the preparation of positive samples to preliminarily determine the quality of phosphorylation-specific antibody

**DOI:** 10.1371/journal.pone.0272138

**Published:** 2022-07-25

**Authors:** Yang Han, Lin Zhong, Fuli Ren

**Affiliations:** 1 Center for Translational Medicine, Wuhan Jinyintan Hospital, Wuhan, Hubei, China; 2 State Key Laboratory of Virology, Wuhan Institute of Virology, Center for Biosafety Mega-Science, Chinese Academy of Sciences (CAS), Wuhan, Hubei, China; 3 Department of Analysis and Reporting, Pfizer (Wuhan) Research and Development Co. LTD, Hubei, China; Indian Institute of Technology Delhi, INDIA

## Abstract

Protein phosphorylation is one of the most common and important post-translational modifications and is involved in many biological processes, including DNA damage repair, transcriptional regulation, signal transduction, and apoptosis regulation. The use of antibodies targeting phosphorylated protein is a convenient method to detect protein phosphorylation. Therefore, high-quality antibodies are essential, and uniform and effective standards are urgently needed to evaluate the quality of these phosphorylation-specific antibodies. In this study, we established a simple, broad-spectrum system for the preparation of phosphorylation-positive samples. The positive samples for evaluation of phosphorylation-specific antibodies were then validated in cells from different species and tissues, and also been proven effectively in western blot, enzyme-linked immunosorbent assays, LC-MS/MS and immunofluorescence analysis. Overall, our findings established a novel approach for evaluation of the quality of phosphorylation-specific antibodies and may have applications in various biomedical fields.

## Introduction

Protein phosphorylation was first discovered in the 1950s [1] and has since been shown to be one of the most common types of intracellular post-translational modification [2,3]. Currently, the fields of protein phosphorylation research include signal transduction [4–7], function of cell membrane [8,9], transcription [10,11], energy metabolism [12–14], and cytoskeletal [14,15] regulation, and reversible protein phosphorylation is thought to be involved in regulation of most aspects of cell life [2]. In simple terms, protein phosphorylation involves the transfer of a phosphate group at the γ site of ATP or GTP to amino acid residues in proteins under the catalytic action of protein kinases [16,17]. Phosphorylation is one of the most important covalent modifications in cells. The reversible process of phosphorylation and dephosphorylation is controlled by protein kinases and phosphatases. To date, over 200,000 phosphorylated sites known to human which site on more than two-thirds of 21,000 human genome encoding proteins have been validated. Furthermore, the human genome also includes approximately 570 protein kinases and 160 protein phosphatases that regulate phosphorylation events [18]. The amino acid residues that are typically subjected to phosphorylation are serine, threonine, and tyrosine; however, aspartic acid, glutamic acid, and cysteine residues may also undergo reversible phosphorylation [19,20].

There are many methods for detecting protein phosphorylation, including isotopic labeling, western blotting, enzyme-linked immunosorbent assay (ELISA), pro-Q Diamond dye, and mass spectrometry [21–25]. Among these methods, western blotting is the most widely used owing to its safety (avoiding the use of isotopes), specificity, and high resolution. Advances in western blotting technology have enabled the production of qualified phosphorylation-specific antibodies to precisely target phosphorylated substrate proteins, providing information on changes in the phosphorylation level of the substrate protein. However, the definition of a qualified anti-phospho-protein antibody has not been established, and all the antibody manufacturers and researchers have reported difficulties in verification of the specificity of anti-phospho-protein antibodies. Indeed, verification of phosphorylation-specific antibodies generally relies on consultation of relevant literature or other data to extract methods for phosphorylation of the corresponding substrate protein. Thus, cells must undergo processing to activate the phosphorylation of the substrate protein, such as overexpression of protein kinases [26], treatment with physical [27] or chemical [28] stimuli, and purification of kinase/substrate proteins [29]. However, the above-mentioned methods for activating phosphorylation have several disadvantages. First, although many studies of phosphorylation have been performed, our understanding of the complex biology of phosphorylation in organisms is still incomplete. Thus, for verification of new phosphorylation-specific antibodies, the appropriate methods for stimulating cells may be unclear. Secondly, even if the phosphorylation of the substrate protein has been activated based on consultation of published literature, the phosphorylation of substrate proteins can still be affected by cell status, cell density, transfection efficiency, stimulus duration, and stimulus concentration. Third, studies of the activation of phosphorylated proteins mainly focused on cells, and it may therefore be difficult to evaluate the effectiveness of phosphorylation-specific antibodies in tissues. Finally, purification of kinase or substrate proteins is a time-consuming and laborious task. Thus, simple and efficient methods for the preparation of phosphorylation-positive samples are urgently needed to verify the phosphorylation-specific antibodies.

Accordingly, in this study, we used a simple and efficient *in vitro* phosphorylation system for the preparation of phosphorylation-positive samples without the need for live cells, kinase and substrate protein purification, or other time-consuming methods. The system could be applied not only in multiple species and tissues but also validated using western blotting, ELISA, and immunofluorescence analysis.

## Materials and methods

### Principles of the *in vitro* phosphorylation system

Phosphorylation of proteins usually refers to the transfer of a phosphate group from ATP to the amino acid side chain of a protein under the catalytic action of enzymes, yielding the phosphorylated protein and ADP. This process is usually reversible via the functions of a kinase, which catalyzes phosphorylation modification, and a phosphatase, which removes the phosphorylation modification. In general, for *in vitro* phosphorylation systems, five factors are considered, i.e., purified kinase, purified substrate protein, reaction buffer, reaction temperature, and ATP. Because kinases and substrate proteins are intrinsically present in in cells, we developed an *in vitro* phosphorylation system using cell lysates instead of purified kinases, purified substrate proteins, and reaction buffer from a traditional protein phosphorylation system.

#### *In vitro* phosphorylation system for western blotting and ELISA

The cell lysate components of the *in vitro* phosphorylation system (*in vitro* buffer) for western blotting and ELISA included 40 mM Tri-HCl (pH 7.5), 10 mM MgCl_2_, 200 μM CaCl_2_, 1 mM DTT, and 1% Triton X-100. The steps for *in vitro* phosphorylation were as follows. After cells or tissues were harvested, cell lysis buffer from the *in vitro* phosphorylation system was added to cells or tissues, and the mixtures were placed in an ice bath for 20 min (tissues were ground using a tissue homogenizer before incubation in the ice bath). The cell lysates were then treated with ultrasound, and supernatants were harvested after centrifugation at 12,000g and 4°C for 15 min. Next, 5 mM ATP (final concentration) was added to the supernatants, and the mixture was placed in a water bath at 30°C for 30 min. Subsequently, the supernatant was used for western blotting and ELISA using standard protocols.

#### *In vitro* phosphorylation system for immunofluorescence analysis

The cell buffer components for the *in vitro* phosphorylation system for immunofluorescence analysis included 40 mM Tri-HCl (pH 7.5), 10 mM MgCl_2_, 200 μM CaCl_2_, 1 mM DTT, 5 mM ATP, and 0.15% Triton X-100. For immunofluorescence, cell morphology is critical for subsequent detection because the nonionic detergent Triton X-100 improves cell membrane permeability and affects cell morphology. In order to maintain the feasibility of *in vitro* phosphorylation and cellular morphology, we explored the optimal concentration of Triton X-100 and found that 0.15% Triton X-100 was most effective. The detailed procedures were as follows. The cells were seeded in immunofluorescent culture dishes and cultured for 24–48 h, and the cell culture medium was then discarded. Next, the cells were washed three times with 1× TBS and incubated with *in vitro* buffer in a carbon dioxide cell incubator at 37°C for 40 min. Subsequently, the *in vitro* buffer was discarded, and the cells were washed three times with 1× TBS. The verification of phosphorylation-specific antibody was then performed using the procedure for immunofluorescence experiments, as described below.

#### Western blotting

Cells were harvested and incubated in *in vitro* buffer with ATP for 30 min, and separated by sodium dodecyl sulfate polyacrylamide gel electrophoresis (SDS-PAGE) on 8–12% gels. Protein concentrations were determined by Bradford assays (Bio-Rad Laboratories, Hercules, CA, USA). After SDS-PAGE, proteins were transferred to polyvinylidene difluoride membranes (Millipore). The membranes were blocked for 1h at room temperature in 5% skim milk and then probed with the indicated primary phosphorylation-specific antibodies at an appropriate dilution overnight at 4°C. The membranes were then incubated with secondary antibodies, and proteins were detected using a Luminescent Image Analyzer (Fujifilm LAS-4000).

#### ELISA

Whole-cell supernatants were harvested using *in vitro* buffer and then evaluated for detection of phosphorylated protein levels using ELISA kits according to the manufacturer’s instructions (Cell Signaling Technology, 7155).

#### Protein digestion

Each sample for mass spectrometry contained about 1×10^7^ 293T cells. After cells were lysated and incubated with or without ATP for 30min at 30°C, urea was added into the lysates with concentration of 8M. The supernatant was collected following centrifugation at 12,000 g at 4°C for 10 min. Then, the mixture was reduced with 5 mM dithiothreitol (DTT) for 45 min at 56°C and alkylated with 15 mM iodoacetamide for 20 min at room temperature in darkness. In the following steps, the mixture was transferred to a 10 kD ultrafiltration tube, 12,000 g at 4°C for 10 min, and the filtrate was discarded. Next, NH_4_HCO_3_ was added to the ultrafiltration tube with the concentration of 25mM, 12,000 g at 4°C for 20 min and discard the filtrate. The filtering steps were repeated for three times. After that, collected proteins were quantified using the Bradford method and digestion was performed by adding trypsin to each sample at 1:50 trypsin to protein mass ratio. Digestion was allowed to proceed overnight at 37˚C and peptides were dissolved in 80% acetonitrile and 2% formic acid prior to phosphorpeptide analysis.

#### Phosphopeptides enrichment

For phosphorylated peptides enrichment, the peptide mixtures were first incubated with immobilized metal affinity chromatography (IMAC)-TiQ_2_ beads with vibration in loading buffer (50% acetonitrile [ACN])/6% trifluoroacetic acid [TFA]) for 30 min. Then the IMAC-TiQ_2_ beads with enriched phosphopeptides were collected by centrifugation, 1,000 g at 4°C for 10 min, then the supernatant was removed. To remove nonspecifically adsorbed peptides, the IMAC microspheres were washed with 50% ACN/6% TFA and 30% ACN/0.1% TFA, sequentially. To elute the enriched phosphopeptides from the IMAC-TiQ_2_, elution buffer containing 10% NH_4_OH was added and the enriched phosphopeptides were eluted with vibration. Then the supernatant was collected and lyophilized for LC-MS/MS analysis.

#### LC-MS/MS

The LC-MS/MS system consisted of a nanoAcquity Ultra Performance LC (UPLC, Ultimate 3000, Thermo Fisher Scientific) coupled with a Q Exactive™ Hybrid Quadrupole-Orbitrap mass spectrometer (Thermo Fisher Scientific). The samples were loaded to an Acclaim PepMap 100 C18 nano-trap column (75μm×2cm, 3μm particles; Thermo Fisher Scientific) using solvent A at a flow rate of 2.5 μL/min for 5min. Peptide separation was then conducted using an Acclaim PepMap RSLC C18 nano-column (75μm×50cm, 2μm particles; Thermo Fisher Scientific). The mobile phase solvent consisted of solvents A and B (0.1% FA in ACN: water [80:20, v/v]), and the flow rate was fixed at 300nL/min. The gradient was set up as follows: solvent B, equilibration at 5% for 15 min, 5–20% for 60 min, 20–50% for 80 min, 50–96% for 1 min, holding at 96% for 10 min, 96–4% for 1 min, and holding at 4% for 17 min for column re-equilibration. The operation parameters were set as follows: the spray voltage was 2.2kV, scan range (m/z) was from 500–2000, resolution of full-MS scan was 60, 000, MS/MS scans at 200 m/z and the resolution was 15, 000. Peptides were selected for tandem mass spectrometry using normalized collision energy (NCE) setting as 28%. Dynamic exclusion was set at 20 s to minimize repeated analyses of the same abundant precursor ions.

#### Identification and quantification of phosphopeptides

Proteome Discoverer (version 2.1) was used to convert the format of the original chromatogram files generated by mass spectrometry, and the data were searched by Mascot (version 2.3) with the human UniProt database. The search parameters were set as follows: Enzyme digestion method was trypsin treatment, which allowed two maximum miss sites. The fixed modification was alkylation of cysteine, and the variable modification was oxidation of methionine, acetylation of protein n-terminal, deamination and phosphorylation of serine, threonine and tyrosine. The precursor mass tolerance was 2.0E-05, and fragment mass tolerances was 0.05 Da. Percolator was used to reprocess the search results of Mascot, and false discovery rate (FDR) ≤ 0.05 was used to filter the trusted peptides.

#### Immunofluorescent assay

Cells were cultured in confocal dishes for 24–48 h and treated with *in vitro* buffer for immunofluorescence analysis. Next, cells were fixed in methanol and acetone (methanol:acetone = 1:1) at 4°C for 20 min. After washing three times with phosphate-buffered saline (PBS) containing Tween (PBST), cells were incubated in 5% bovine serum albumin (BSA) in PBST at room temperature for 30 min and then incubated with phosphorylation-specific antibodies (1:100) for 4 h at room temperature. After washing in PBST three times, cells were incubated with Cy3-conjugate secondary antibodies for 45 min at 37°C. Cells were then washed three times with PBST, incubated with 4′,6-diamidino-2-phenylindole(DAPI) for 5 min at 37°C, and washed again with methanol three times and PBS three times. Finally, cells were analyzed using a confocal laser-scanning microscope (Olympus).

#### Cells and tissues

The basic information of all cell lines used in this study are described as follows: 293T (Human embryonic kidney cell line), Jurkat (Human T-acute lymphoblastic leukemia cell line), Vero (African green monkey kidney cell line), PC-12 (Rat Adrenal gland pheochromocytoma cell line), L929 (mouse fibroblast cell line), C2C12 (mouse myoblast cell line) and C6 (Rat glioma cell line). All types of cells were purchased from China Center for Type Culture Collection (CCTCC) and were cultured according to the instructions provided by the institute. And all kinds of cell lines were identified by CCTCC with STR (short tandem repeat) authentication. In addition, mice which provided tissues were obtained from the animal housing facility of the Chinese Academy of Sciences (Changsha, China). Based on “Guideline for Animal Care and Use, Wuhan Institute of Virology (WIV), Chinese Academy of Sciences (CAS)”, the Institutional Review Board (IRB) at WIV, CAS approved all protocols for animal experiments in this study. All processes in our study with tissues from mice were preformed according to ethical standards for research using animals.

### Statistical analysis

All statistical analysis were performed with GraphPad Prism software version 8, and P value less than 0.05 was considered statistically significant.

## Results

### Feasible and ATP-dependent *in vitro* phosphorylation system

**Fig 1:**
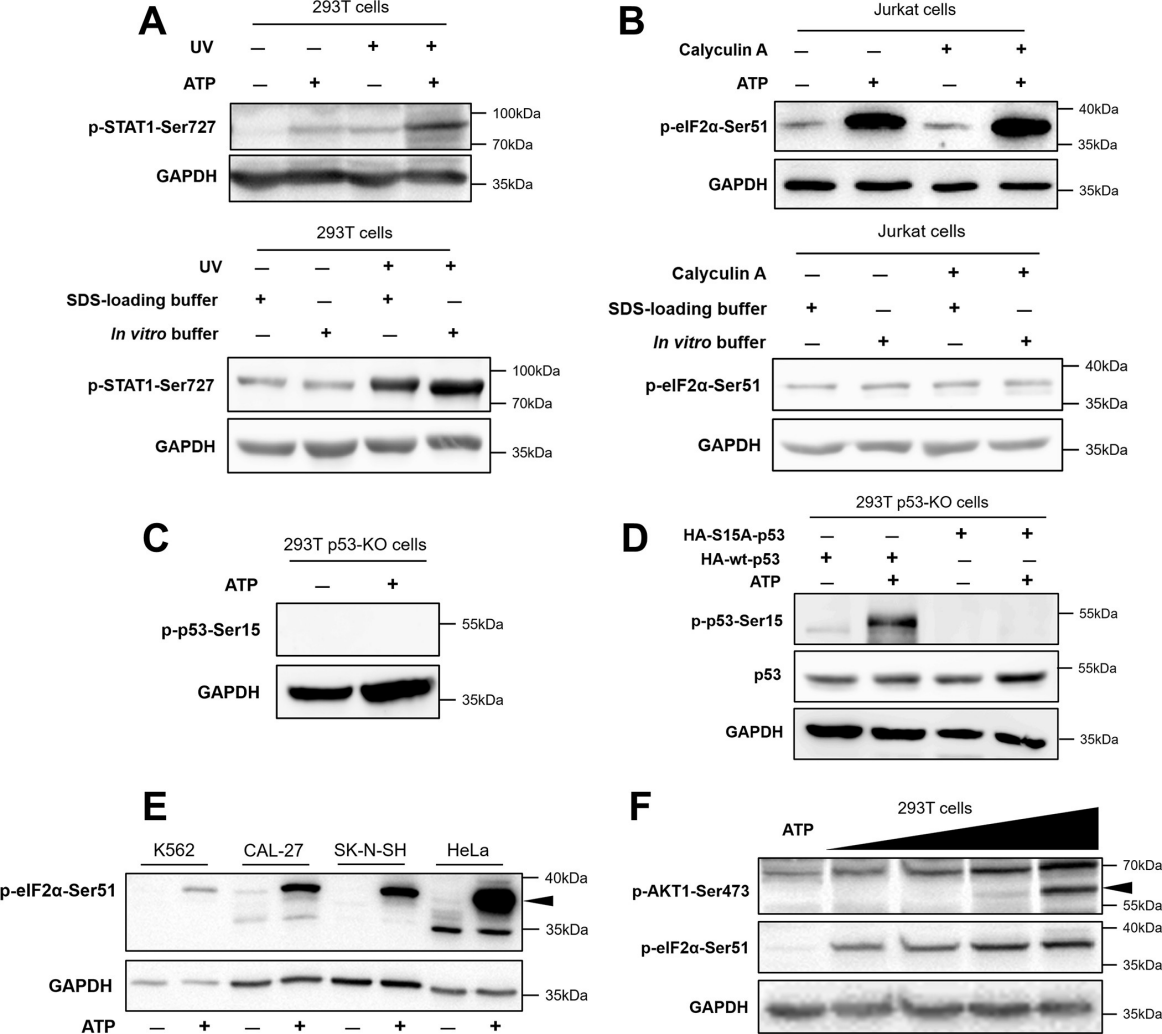
Development of a feasible, ATP-dependent *in vitro* phosphorylation system. (A) Upper panel: 293T cells were first treated with or without UV-A irradiation for 30 min, and cells were then harvested and lysed in *in vitro* buffer. Lysates were incubated with or without 5 mM ATP for 30 min. Phospho-STAT1 (Ser727) was detected by western blotting. Lower panel: 293T cells with or without UV-A treatment were lysed by *in vitro* buffer or SDS-loading buffer, then Phospho-STAT1 (Ser727) was detected by western blotting. (B) Upper panel: Jurkat cells were first treated with or without calyculin A (100 nM) for 30 min, and cells were then harvested and lysed in *in vitro* buffer. Next, lysates were incubated with or without 5 mM ATP for 30 min. Phospho-eIF2α (Ser51) was detected by western blotting. Lower panel: Jurkat cells with or without calyculin A treatment were lysed by *in vitro* buffer or SDS-loading buffer, then phospho-eIF2α (Ser51) was detected by western blotting. (C) The p53-KO 293T cells were harvested and lysed in *in vitro* buffer. Then lysates were incubated with or without 5 mM ATP for 30 min. (D) The p53-KO 293T cells were transfected with wt-p53 or S15A-p53 for 24h, after that cells were lysed in *in vitro* buffer and then incubated with or without 5 mM ATP for 30 min. (E) K562, CAL-27, SK-N-SH, and HeLa cells were harvested, lysed in *in vitro* buffer, and then incubated with or without 5 mM ATP for 30 min. Phospho-eIF2α (Ser51) was detected by western blotting. (F) 293T cells were harvested, lysed in in vitro buffer, and incubated with 0, 0.5, 1, 2, or 5 mM ATP for 30 min. Phospho-eIF2α (Ser51) and phospho-AKT1 (Ser473) were detected by western blotting.

To verify the feasibility of our system, we first activated and detected phosphorylated proteins using human 293T and Jurkat cells. 293T cells were first treated with or without ultraviolet (UV) light and then incubated with *in vitro* buffer, ATP was added as needed. Besides that, 293T cells with or without UV treatment were also directly lysed into the SDS-PAGE loading samples by *in vitro* buffer or SDS-loading buffer. The results showed that phosphorylated STAT1 (Ser727) was activated by UV light and ATP, and here ATP acted as a stronger phosphorylation activator than UV light. Meanwhile, the phosphorylation level of STAT1 in cells not incubated with ATP^+^ *in vitro* buffer failed activating compared with SDS-loading buffer (Fig 1A). In another examples, calyculin A as a phosphatase inhibitor is widely used in phosphorylation enhancement of substrate proteins including eIF2α. Antibody manufacturers usually utilize calyculin A to activate the phosphorylation of eIF2α for validating the availability of anti-phospho-eIF2α (Ser51). However, the stimuli often fail to activate phosphorylation of substrate protein due to some factors such as concentration and duration of stimulation. In our study, the phosphorylation sites in Jurkat cells were activated by calyculin A or ATP with *in vitro* buffer, and we also found that calyculin A did not activate the phosphorylation of eIF2α (Ser51). Not surprisingly, neither *in vitro* buffer nor SDS-loading buffer could make eIF2α (Ser51) phosphorylation after induced by calyculin A. The results showed that the *in vitro* phosphorylation system could activate eIF2α phosphorylation efficiently even when the stimulus didn’t work (Fig 1B). To explore whether *in vitro* phosphorylation system affect the recognition specificity of phosphorylated antibodies, we further validated the reaction system in 293T cells with p53 knocked out (p53-KO 293T cells, homozygous). The results showed that the p53 phosphorylation (ser15) could not be detected or activated, even if ATP were added (Fig 1C). Then, wild-type p53 (wt-p53) and phosphorylated site mutant p53 (S15A-p53) were transfected into p53-KO 293T cells, respectively. After that, ATP was added or not to find out the variation trend of p53 phosphorylation. As a result, the phosphorylation of p53 was activated only after the wt-p53 were replenished into p53-KO cells and incubated with ATP (Fig 1D). The feasibility of the *in vitro* phosphorylation system was then verified in K562, CAL-27, SK-N-SH, and HeLa cells (Fig 1E). The phosphorylation of eIF2α (Ser51) in all cell types was activated by ATP in our *in vitro* phosphorylation system. Further investigation of the ATP dependence of the system using 0, 0.5, 1, 2, and 5 mM ATP showed that the phosphorylation of AKT1 (Ser473) and eIF2α (Ser51) occurred in an ATP concentration-dependent manner (Fig 1F).

### Application of the *in vitro* phosphorylation system in other species and tissues

**Fig 2:**
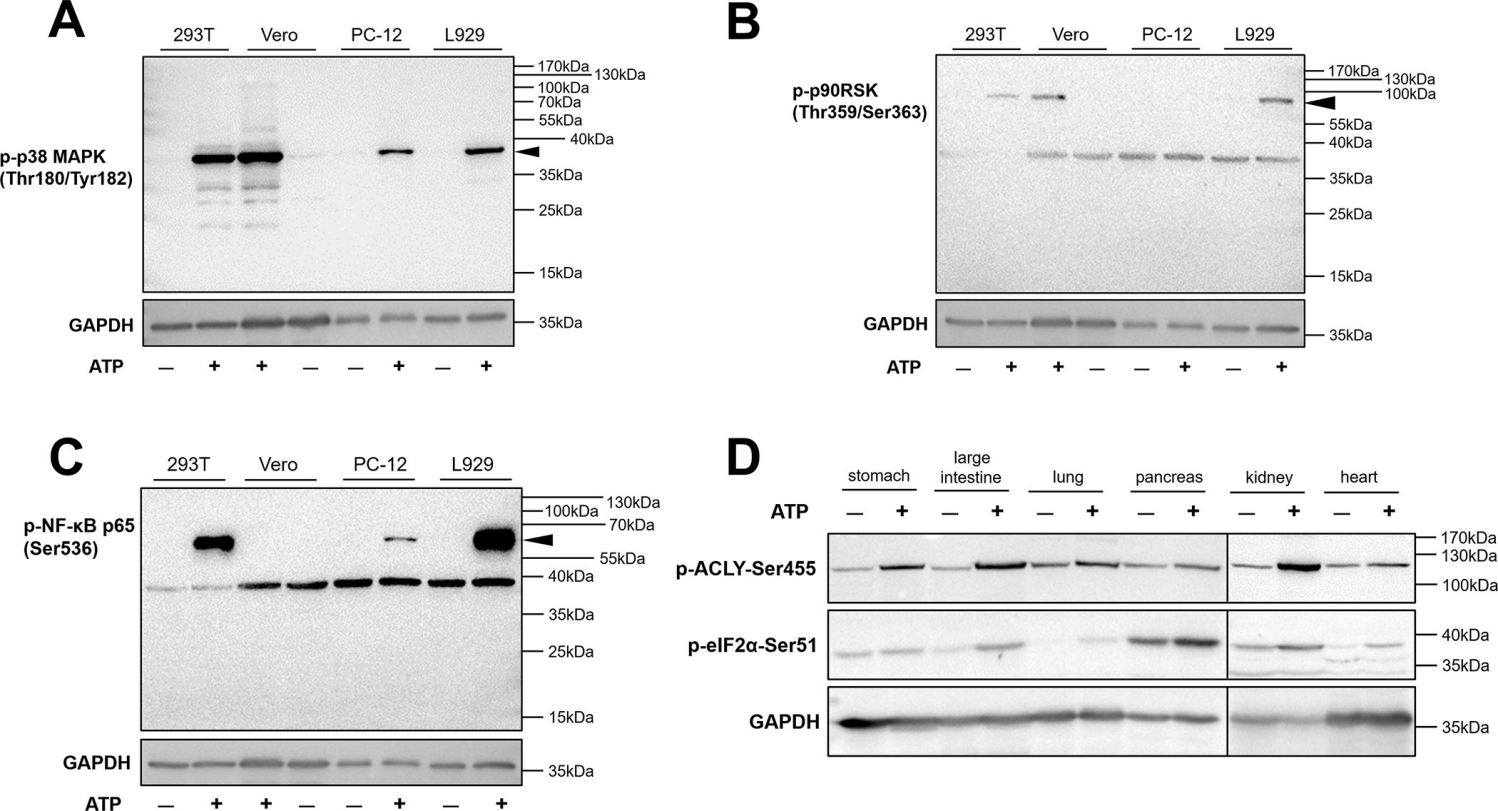
Application of the *in vitro* phosphorylation system to different types of cells and tissues. (A–C) 293T, Vero, PC-12, and L929 cells were cultured for 24 h, harvested, and then incubated with or without 5 mM ATP in an in *in vitro* buffer for 30 min. Phospho-p38 MAPK (Thr180/Tyr182), phospho-p90RSK (Thr359/Ser363), and phospho-NF-κB-p65 (Ser536) were detected in cells by western blotting. (D) Stomach, large intestine, lung, pancreas, kidney, and heart tissues from mice were ground in *in vitro* buffer and incubated with or without 5 mM ATP for 30 min. Phospho-ACLY (Ser455) and phospho-eIF2α (Ser51) were detected by western blotting.

To explore the feasibility of our *in vitro* phosphorylation system in cells from other species, we assessed 293T, Vero, PC-12, and L929 cells from humans, monkeys, rats, and mice, respectively. In the *in vitro* phosphorylation system, all cell types were treated with or without ATP, and phospho-p38 mitogen-activated protein kinase (MAPK; Thr180/Tyr182), phospho-p90RSK (Thr359/Ser363), and phospho-nuclear factor (NF)-κB-p65 (Ser536) were detected by western blotting (Fig 2). The results showed that phospho-p38 MAPK (Thr180/Tyr182) was detectable using antibodies in all cell types (Fig 2A). Additionally, phospho-p90RSK (Thr359/Ser363) was detectable in 293T, Vero, and L929 cells (Fig 2B), and phospho-NF-κB-p65 (Ser536) was detectable in 293T, PC-12, and L929 cells (Fig 2C). Thus, these findings indicated that our *in vitro* phosphorylation system was also applicable to species other than humans.

Next, we evaluated whether our system could activate phosphorylation in tissues. In our study, different mouse tissues were used for verification of phosphorylation-specific antibodies. The results showed that tissue samples from the stomachs, large intestines, lungs, pancreases, kidneys, and hearts of mice were phosphorylated in our *in vitro* phosphorylation system; phosphorylation of ATP citrate lyase (ACLY) and eIF2α was significantly activated, as demonstrated by western blotting (Fig 2D). According to these results, phosphorylation of substrate proteins by the *in vitro* phosphorylation system met the validation requirements for multiple species and tissues. This method has no effect on the recognition specificity of anti-phospho-protein antibodies and could be used to determine the detection scope of the phosphorylation-specific antibodies in a simple, rapid manner.

### Phosphorylation can be activated by GTP

**Fig 3:**
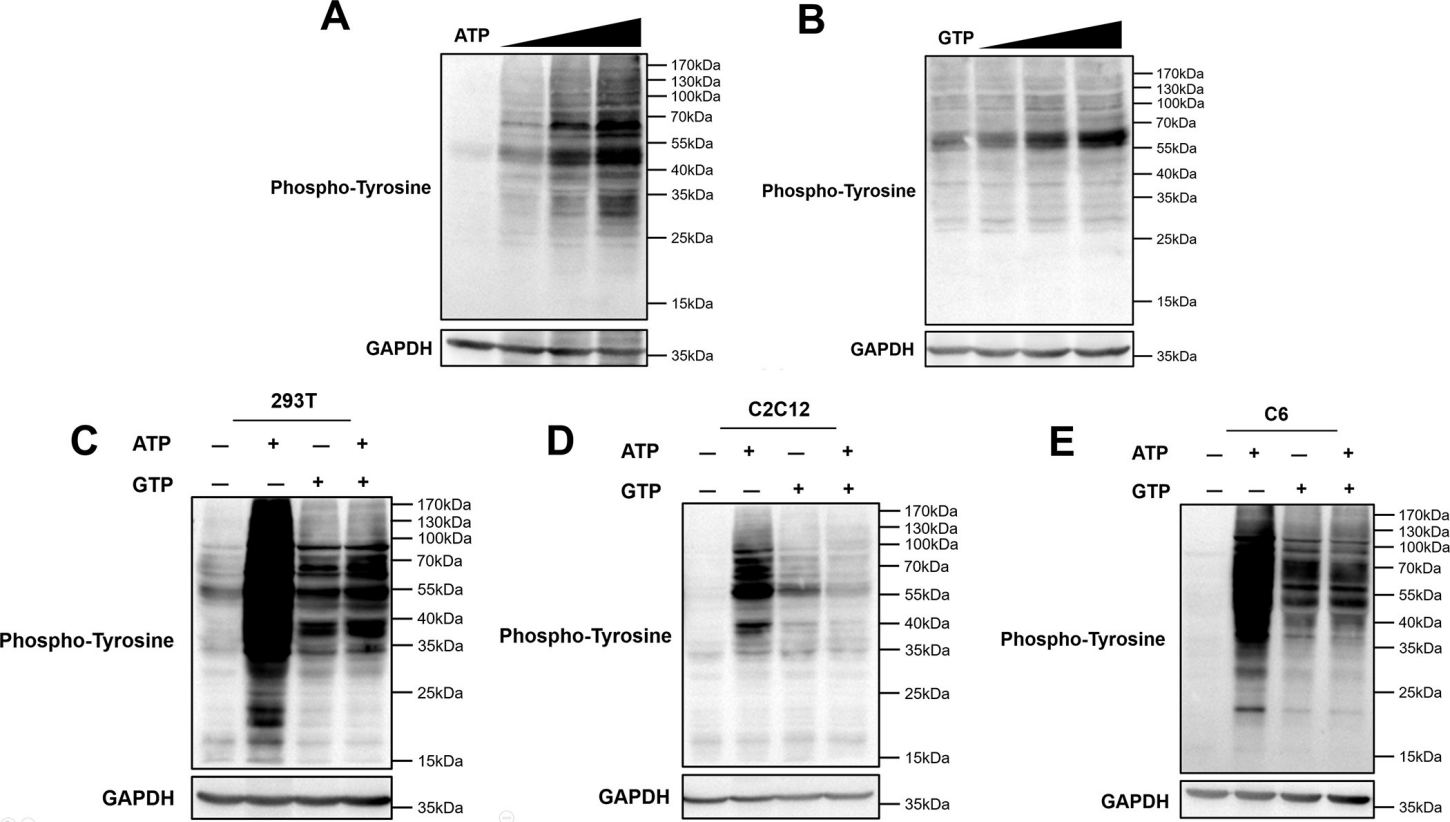
Phosphorylation was activated by GTP in our *in vitro* phosphorylation system. (A, B) 293T cells were cultured for 24 h. Cells were then harvested and incubated with increasing concentrations of ATP or GTP (0, 1, 2.5, and 5 mM) for 30 min at 30°C. Anti-phospho-tyrosine antibodies were used for detection of total tyrosine phosphorylation by western blotting. (C-E) 293T, C2C12, and C6 cells were cultured, harvested, and then incubated with 5 mM ATP, 5 mM GTP, or 5 mM ATP plus 5 mM GTP for 30 min at 30°C. Anti-phospho-tyrosine antibodies were then used for detection of total tyrosine phosphorylation by western blotting.

In addition to ATP, GTP can also transfer phosphate groups to substrate proteins. Therefore, to explore the roles of ATP and GTP in our *in vitro* phosphorylation system, different concentrations of ATP and GTP were added to 293T cell lysates (final concentrations of 0, 1, 2.5, and 5 mM; Fig 3A and 3B). Subsequently, phosphorylation-specific antibodies were used to detect total tyrosine phosphorylation. The results showed that both ATP and GTP enhanced total tyrosine phosphorylation levels. Next, 5 mM ATP or GTP was added individually or in combination to our *in vitro* phosphorylation system using 293T, C2C12, and C6 cells (Fig 3C–3E). We found that individual supplementation with ATP or GTP enhanced the levels of total tyrosine phosphorylation. Notably, ATP activated more phosphorylation sites on substrate proteins than GTP. That might be because most phosphorylated proteins in cells derive their phosphate groups from ATP. Interestingly, when ATP and GTP were added to the system simultaneously, total phosphorylation did not increase but was lower than that with ATP individual supplementation.

### Applicability of the system for ELISA and LC-MS/MS

**Fig 4:**
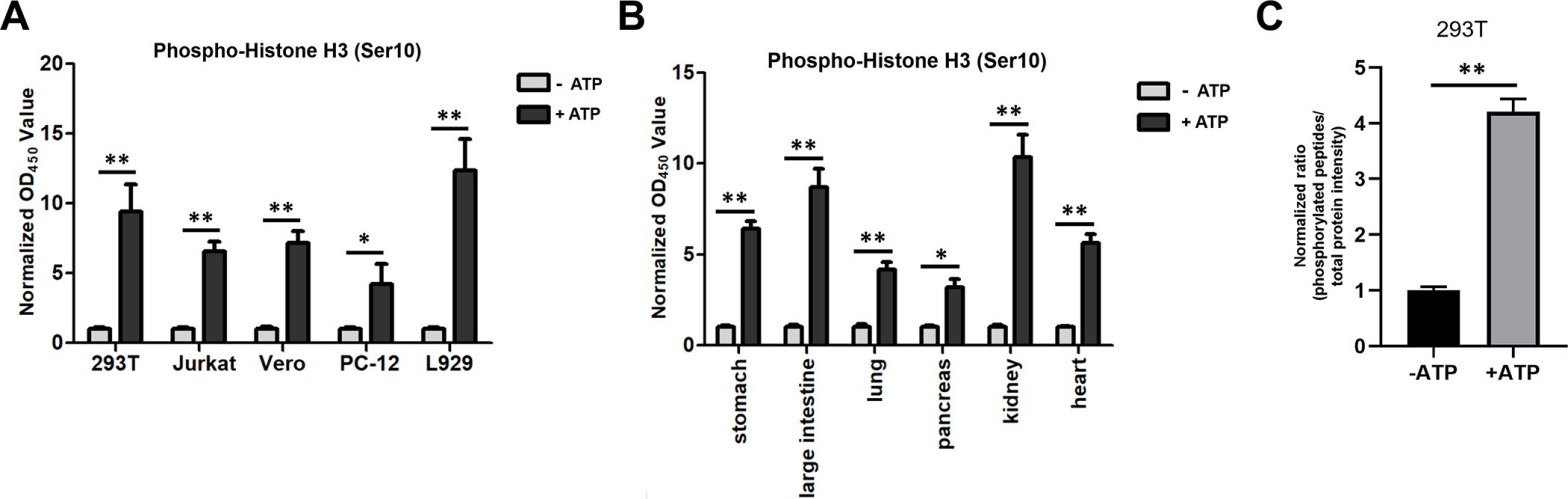
Application of our *in vitro* phosphorylation system for ELISA and LC-MS/MS. (A) 293T, Jurkat, Vero, PC-12, and L929 cells were cultured, harvested, and lysed in *in vitro* buffer. Cell lysates were then incubated in the phosphorylation system with or without 5 mM ATP. After centrifugation, the supernatants were used for ELISA with anti-phospho-histone H3 (Ser10) antibodies. (B) Stomach, large intestine, lung, pancreas, kidney, and heart tissues from mice were ground and lysed, and supernatants were incubated in *in vitro* buffer with or without 5 mM ATP. The supernatants were then applied for ELISA with anti-phospho-histone H3 (Ser10) antibodies. (C) Each cell sample contained about 1×107 293T cells, then cells were lysed and incubated with or without ATP for 30min at 30°C. After that, proteins in lysates were conducted with digestion, phosphorylated peptides enrichment and LC-MS/MS to calculate the ratio of phosphopeptides to total protein intensity.

Above results indicated that our *in vitro* phosphorylation system could be applied to western blotting experiments to increase the phosphorylation levels of substrate proteins in cells or tissues. Subsequently, we explored whether the system could be used in the verification of phosphorylation-specific antibodies in ELISA using supernatants from 293T, Jurkat, Vero, PC-12, and L929 cells (Fig 4A) and anti-phospho-histone H3 (Ser10) antibodies. The results showed that the OD_450_ values corresponding to phosphorylation of histone H3 (Ser10) were significantly increased in all cell lysates.

Next, we explored whether tissues incubated with the *in vitro* phosphorylation system could be used for detection of phosphorylation using ELISA. Supernatants of stomach, large intestine, lung, pancreas, kidney, and heart tissues from mice were incubated in the *in vitro* phosphorylation system with or without ATP (Fig 4B) and were then used in ELISAs with anti-phospho-histone H3 (Ser10) antibodies. We found that the *in vitro* phosphorylation system could activate phosphorylation of substrate proteins and could be used to identify whether phosphorylation-specific antibodies were suitable for analysis of phosphorylated protein levels in tissues and cells by ELISA.

To further explore the effect of *in vitro* phosphorylation system on the variation of intracellular phosphorylated protein abundance, 293T cells were lysed and incubated with or without ATP for 30 min at 30°C. Then the lysates were treated by protein digestion, phosphopeptide enrichment and LC-MS/MS to determine the variation ratio of phosphorylated peptides to protein intensity. The results demonstrated that the *in vitro* phosphorylation system with ATP could significantly increase the proportion of phosphorylated peptides (Fig 4C; *: *P*<0.01, **: *P*<0.001).

### Applicability of the system in immunofluorescence analyses

**Fig 5:**
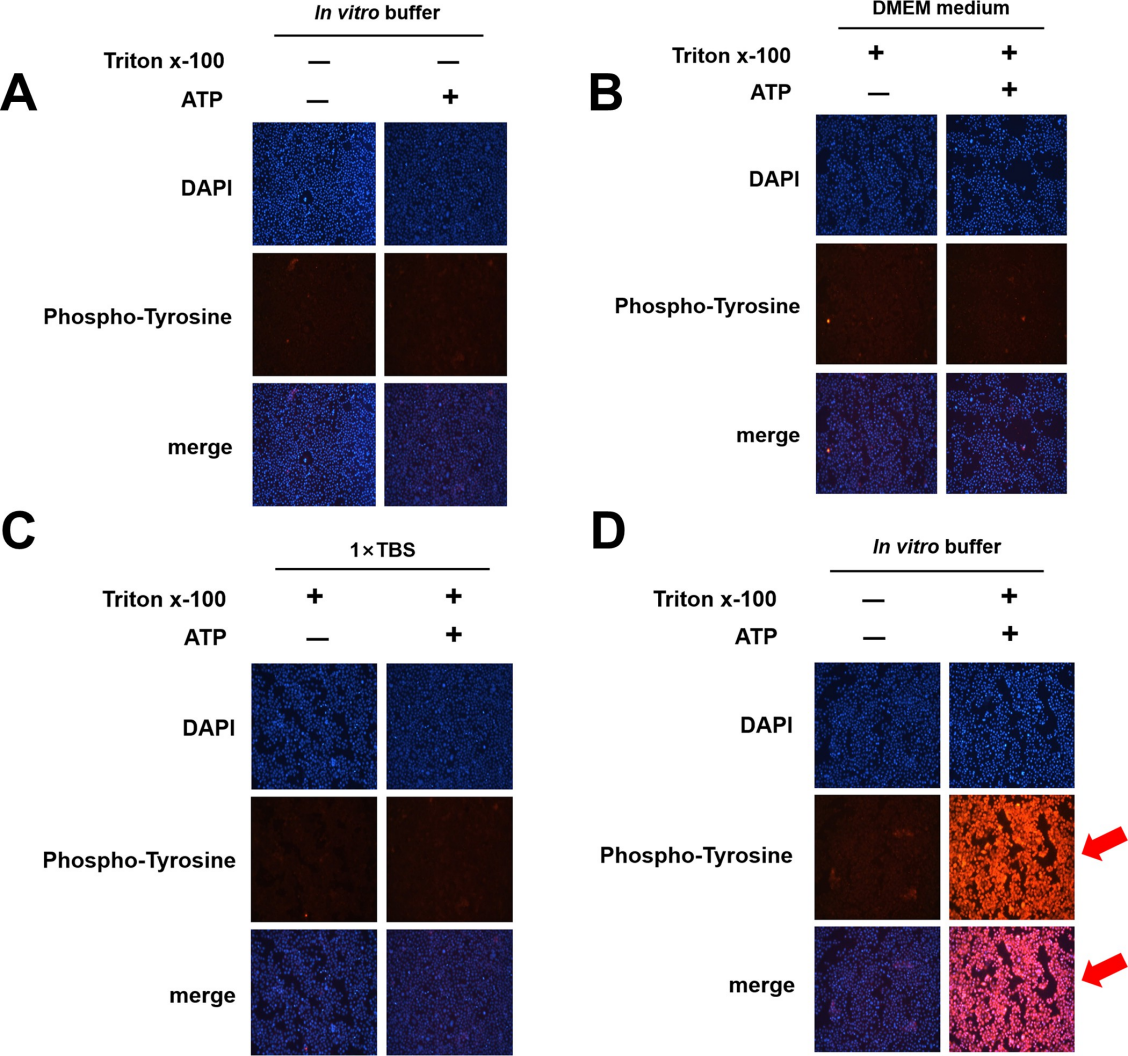
Application of our *in vitro* phosphorylation system for immunofluorescence. 293T cells were cultured for 24 h. Next, cells were incubated with (A) *in vitro* buffer with or without 5 mM ATP at 37°C for 40 min; (B) DMEM with or without 5 mM ATP and 0.15% TritonX-100 at 37°C for 40 min; (C) 1× TBS medium with or without 5 mM ATP and 0.15% TritonX-100 at 37°C for 40 min; or (D) *in vitro* buffer with or without 5 mM ATP and 0.15% TritonX-100 at 37°C for 40 min. Subsequently, tyrosine phosphorylation in cells was detected with anti-phospho-tyrosine antibodies using immunofluorescence analysis.

In order to further evaluate the application scope of our *in vitro* phosphorylation system, we tested the feasibility of this system in immunofluorescence experiments. Initially, 293T cells were cultivated for 24 h, and the medium was replaced with *in vitro* buffer. Cells were then incubated with or without ATP at 37°C for 40 min. Tyrosine phosphorylation was then detected using anti-phospho-tyrosine antibodies in immunofluorescence analysis. The results showed no increase in tyrosine phosphorylation levels (Fig 5A). Thus, we assumed that the cells had membrane barriers that prevented the *in vitro* buffer or ATP from entering the cells efficiently. Accordingly, we added 0.15% TritonX-100 to the reaction system to increase membrane permeability. In addition, to verify the effectiveness of the *in vitro* phosphorylation system, 1× TBS and Dulbecco’s modified Eagle’s medium (DMEM) were added to the cells as controls (Fig 5B–5D), and cells were incubated with or without ATP at 37°C for 40 min. The total degree of tyrosine phosphorylation was detected with anti-phospho-tyrosine antibodies using immunofluorescence analysis. The results demonstrated that the addition of ATP did not activate phosphorylation of substrate proteins in 1× TBS or DMEM, but significantly enhanced tyrosine phosphorylation levels when using *in vitro* buffer.

## Discussion

In this study, we developed an *in vitro* phosphorylation system to phosphorylate efficiently and simply intracellular substrate proteins with intrinsic substrate proteins and protein kinases. The system was capable of extensive activation of phosphorylation in cells from a variety of species. Although phosphorylation is generally difficult to activate in tissue samples, we achieved phosphorylation of substrate proteins in tissue samples using our system. Moreover, we demonstrated that the system could be used for evaluation of phosphorylation-specific antibodies in a variety of experimental methods, including western blotting, ELISA, and immunofluorescence analysis and the system may also have application value in other experimental systems. This universal phosphorylation activation method effectively ensured the phosphorylation of substrate proteins, thereby enhancing the credibility of phosphorylation-specific antibody verification.

Currently, phosphorylation of substrate proteins is generally activated via three approaches. First, plasmids encoding protein kinases or substrate proteins can be transfected into cells; however, the transfection efficiency is generally low owing to the different characteristics of various cell lines. Second, phosphorylation in cells can be activated by physical or chemical stimuli or bioactivators. This approach is limited by the concentration, duration and temperature of the reaction, which may lead to unreliable effects. Third, kinases and substrate proteins can be purified from eukaryotic or prokaryotic expression systems, and phosphorylation of purified substrate proteins can be activated by purified substrate proteins *in vitro*. This method can guarantee the specificity of protein phosphorylation, but it is time-consuming and costly, and the purified kinases and substrate proteins have to show biological activity.

**Fig 6:**
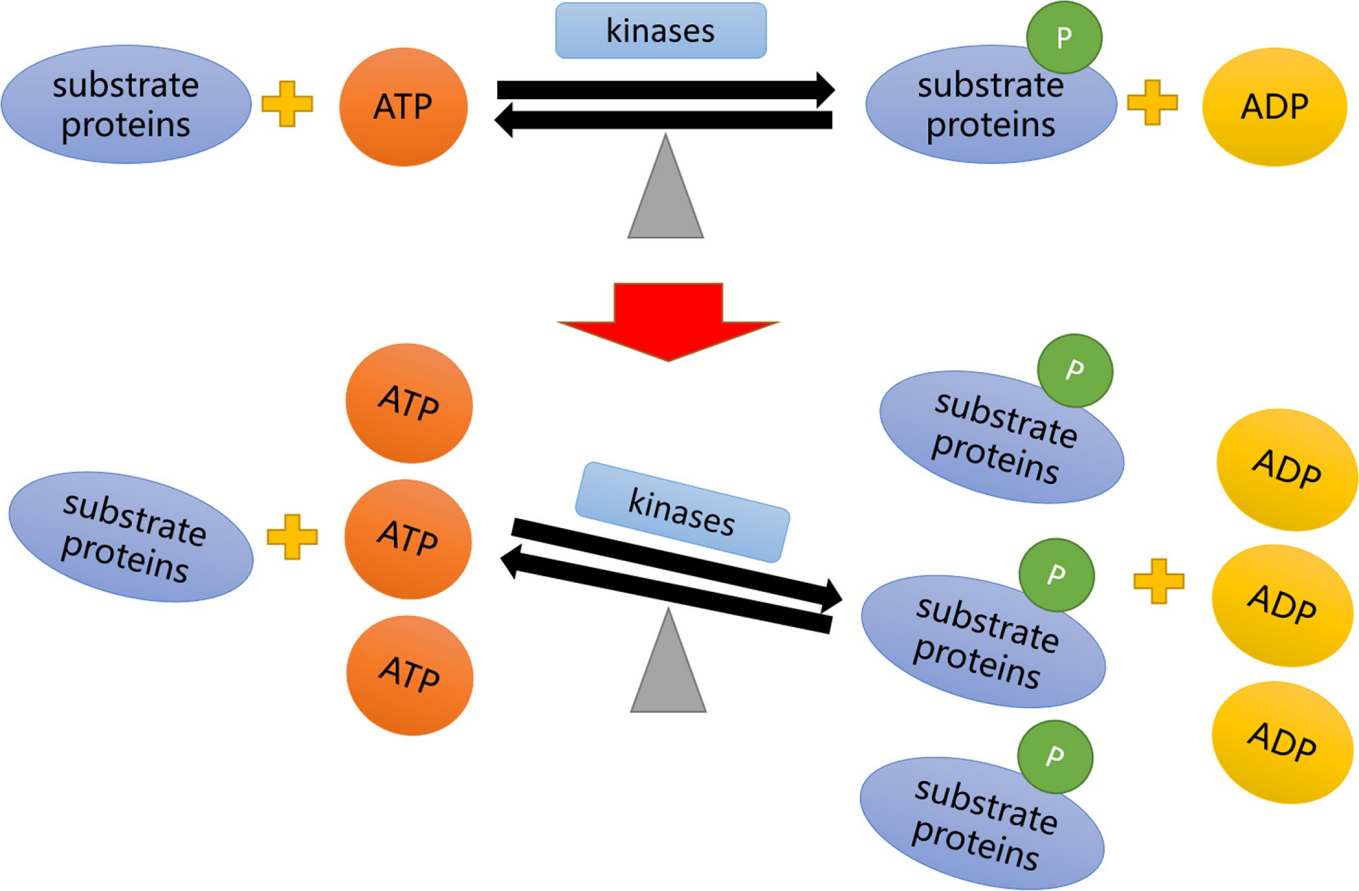
Schematic diagram of *in vitro* phosphorylation system.

The principle of our *in vitro* phosphorylation system is simple, and the approach displays effectiveness for various cell lines and tissues. No kinases or substrate proteins was added in the system; instead, all the components were endogenous proteins expressed in the cells. The addition of ATP could shift the chemical equilibrium, leading to the phosphorylation of lots of substrate proteins (Fig 6). In our study, this *in vitro* phosphorylation system was successfully applied to detect the phosphorylation levels in various cells (293T, Jurkat, K562, CAL-27, SK-N-SH, HeLa, Vero, PC-12, L929, C2C12, and C6 cells) from different species (humans, monkeys, rats, and mice). In addition, activation of protein phosphorylation under this system was not accidental, and we have demonstrated this by measuring the level of total tyrosine phosphorylation. Furthermore, this *in vitro* phosphorylation system was applicable at the tissue level and could be used to evaluate phosphorylation in the stomach, large intestine, lung, pancreas, kidney, and heart, thereby establishing a novel approach to evaluation of the applicability of anti-phospho-protein antibodies at the tissue level. Quality detection of anti-phospho-protein antibodies for ELISA and immunofluorescence analysis could also be performed using the *in vitro* phosphorylation system. Therefore, the system provides an efficient, simple approach for the screening of high-quality, versatile anti-phospho-protein antibodies.

Despite there are lots of advantages, our system still has one limitation. We believed that *in vitro* phosphorylation systems can be used for screening of anti-phospho-protein antibodies, e.g., by flow cytometry and immunohistochemistry; however, we have not yet evaluated these approaches. Therefore, in future studies, we will perform additional analyses of this topic.

In summary, we developed an *in vitro* system that enabled extensive phosphorylation of proteins in a variety of cells and tissues. This system showed feasibility in determining the quality of anti-phospho-protein antibodies. Phosphorylated substrate proteins can be evaluated by western blotting, ELISA, and immunofluorescence analysis, and our system could theoretically be applied in many other experimental scenarios. This system may greatly improve the screening efficiency of phosphorylated antibodies and reduce the costs associated with the use of substantial chemical, physical, and biological stimuli. Finally, this system is expected to facilitate the study of protein phosphorylation by both antibody manufacturers and researchers all over the world.

## Supporting information

S1 Table OD values of phosphor-Histone H3-Ser10. OD values of phosphor-Histone H3-Ser10 in different tissues and cells were proceeded by ELISA assay after incubated in *in vitro* phosphorylation system with or without ATP.
(XLSX)

S2 Table Number of phosphorylated peptides and total protein intensities. The lysates of 293T cells were incubated in *in vitro* phosphorylation system with or without ATP. Then the number of phosphorylated peptides and total protein intensities were analyzed by LC-MS/MS.
(XLSX)

S1 Raw images (PDF)
