# Supplementary material for: A simple method for the preparation of positive samples to preliminarily determine the quality of phosphorylation-specific antibody
Source: PLoS One. 2022 Jul 25;17(7):e0272138. doi: 10.1371/journal.pone.0272138 (PMC9312364; doi:10.1371/journal.pone.0272138)

Fig. 1A
Upper panel

The proteins were detected using a Luminescent Image Analyzer (Fujifilm LAS-4000)

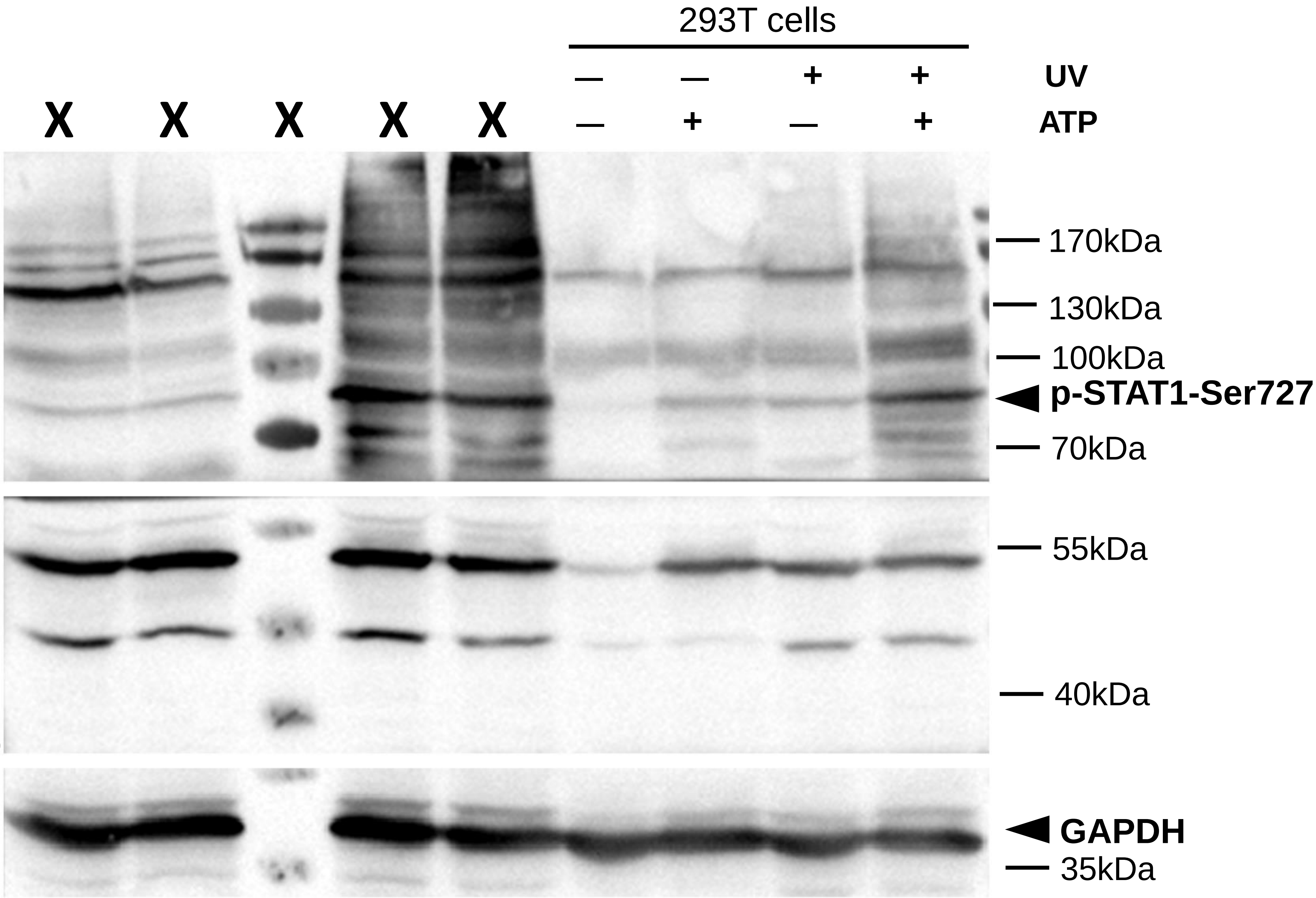

Fig. 1A
Lower panel

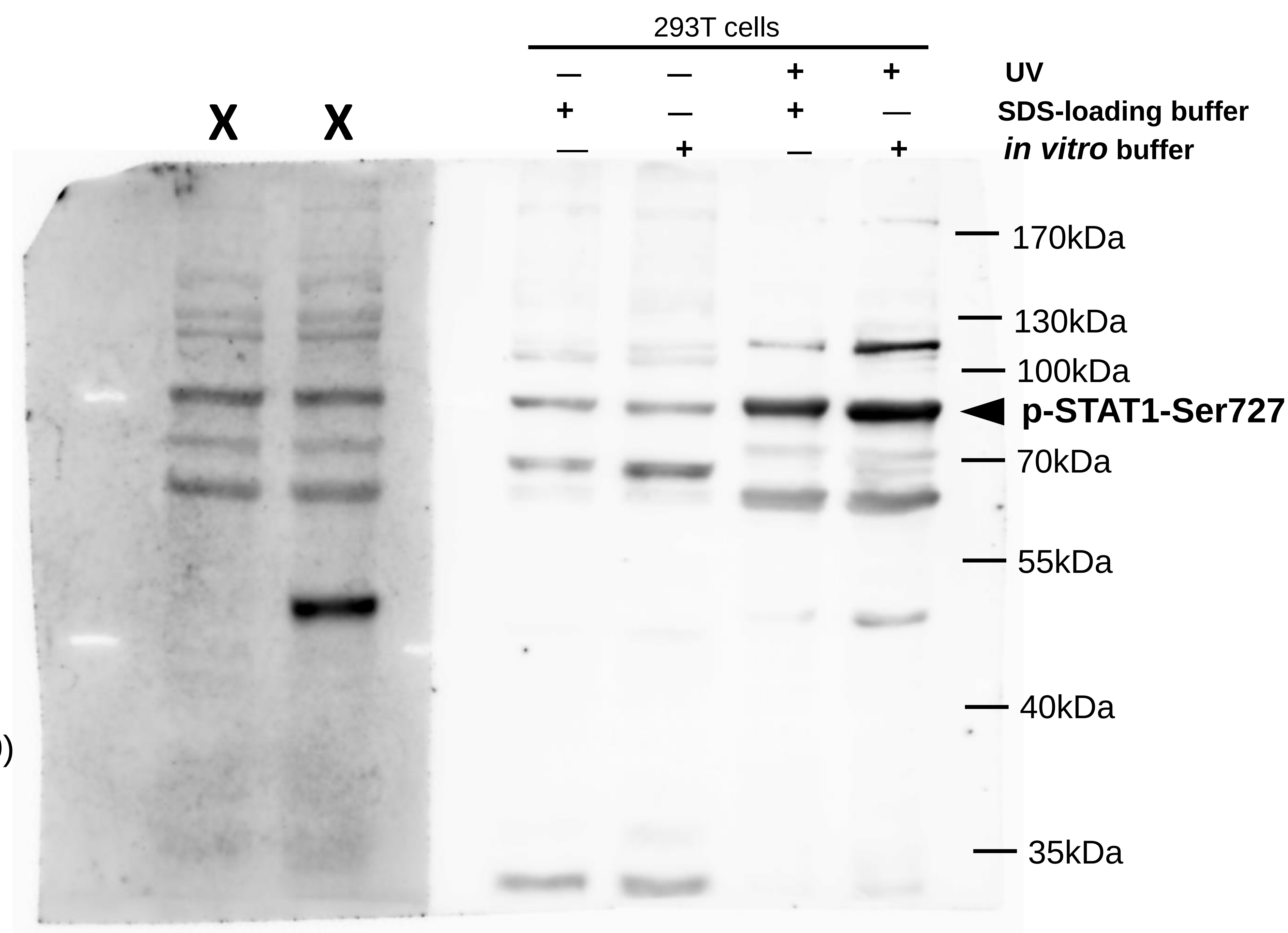

The proteins were detected using a Luminescent Image Analyzer (Fujifilm LAS-4000)

Fig. 1A
Lower panel

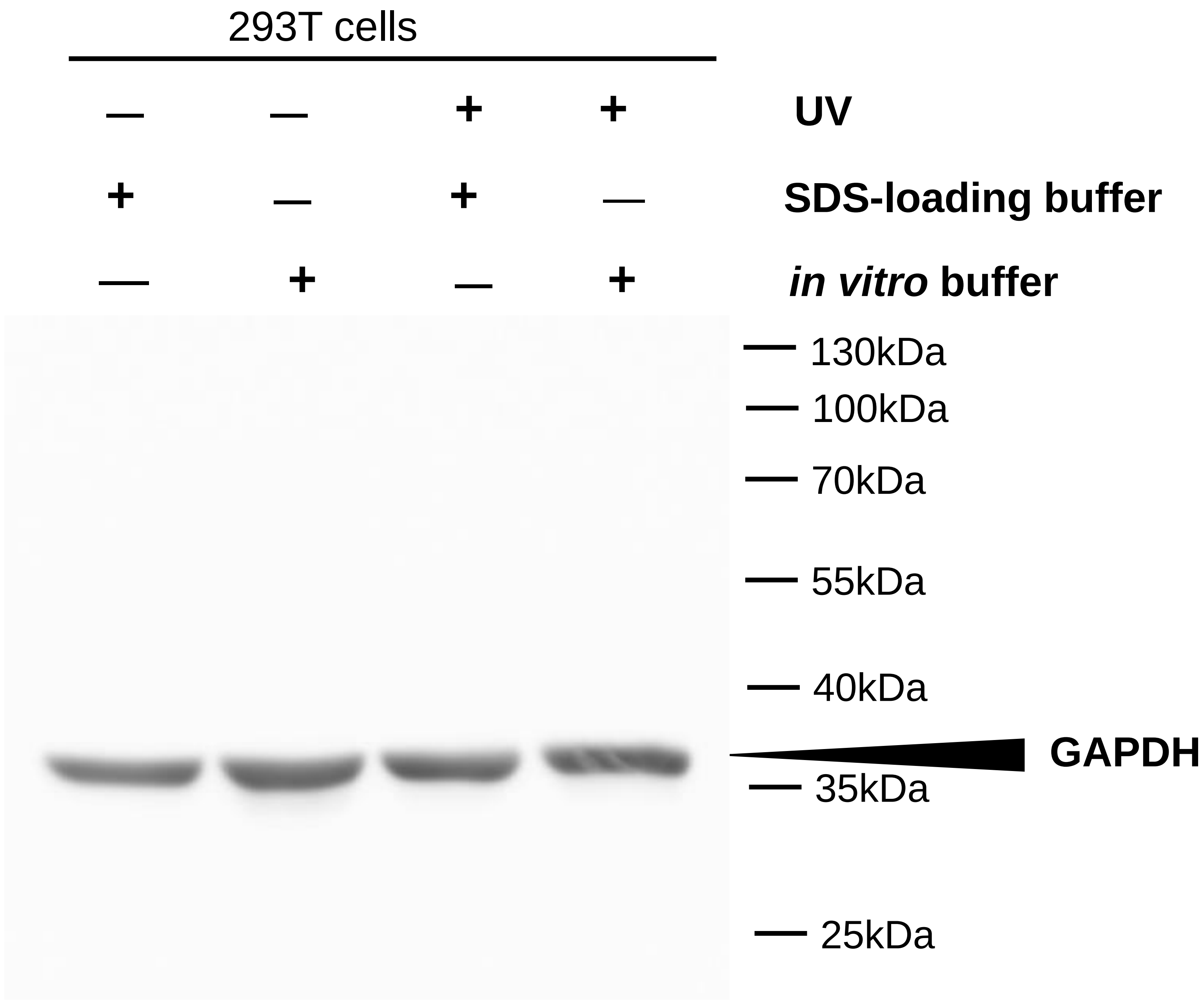

The proteins were detected using a Luminescent Image Analyzer (Fujifilm LAS-4000)

Fig. 1B
Upper panel

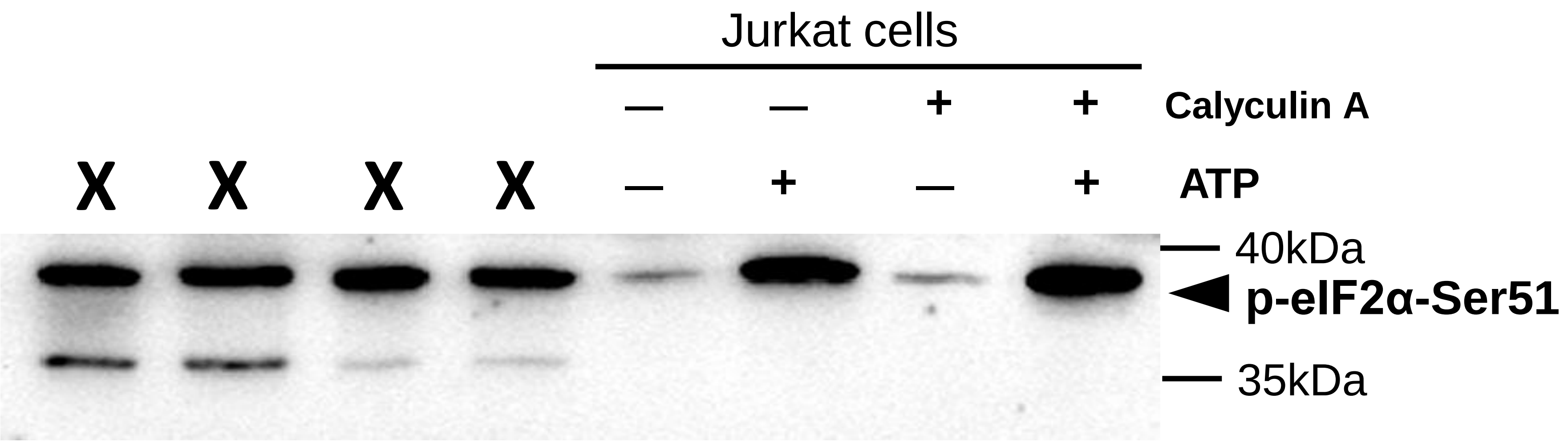

The proteins were detected using a Luminescent Image Analyzer (Fujifilm LAS-4000)

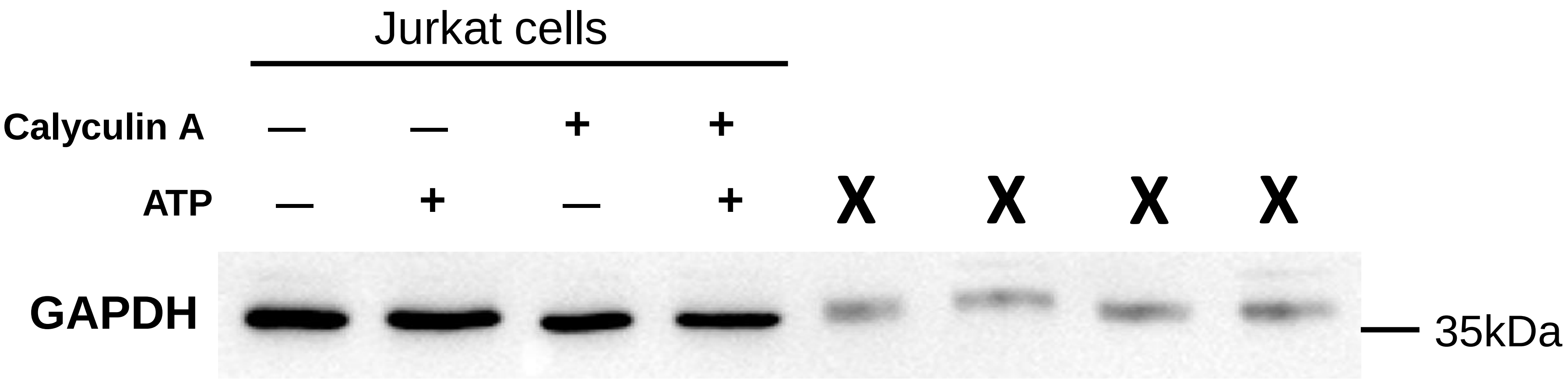

Fig. 1B
Lower panel

The proteins were detected using a Luminescent Image Analyzer (Fujifilm LAS-4000)

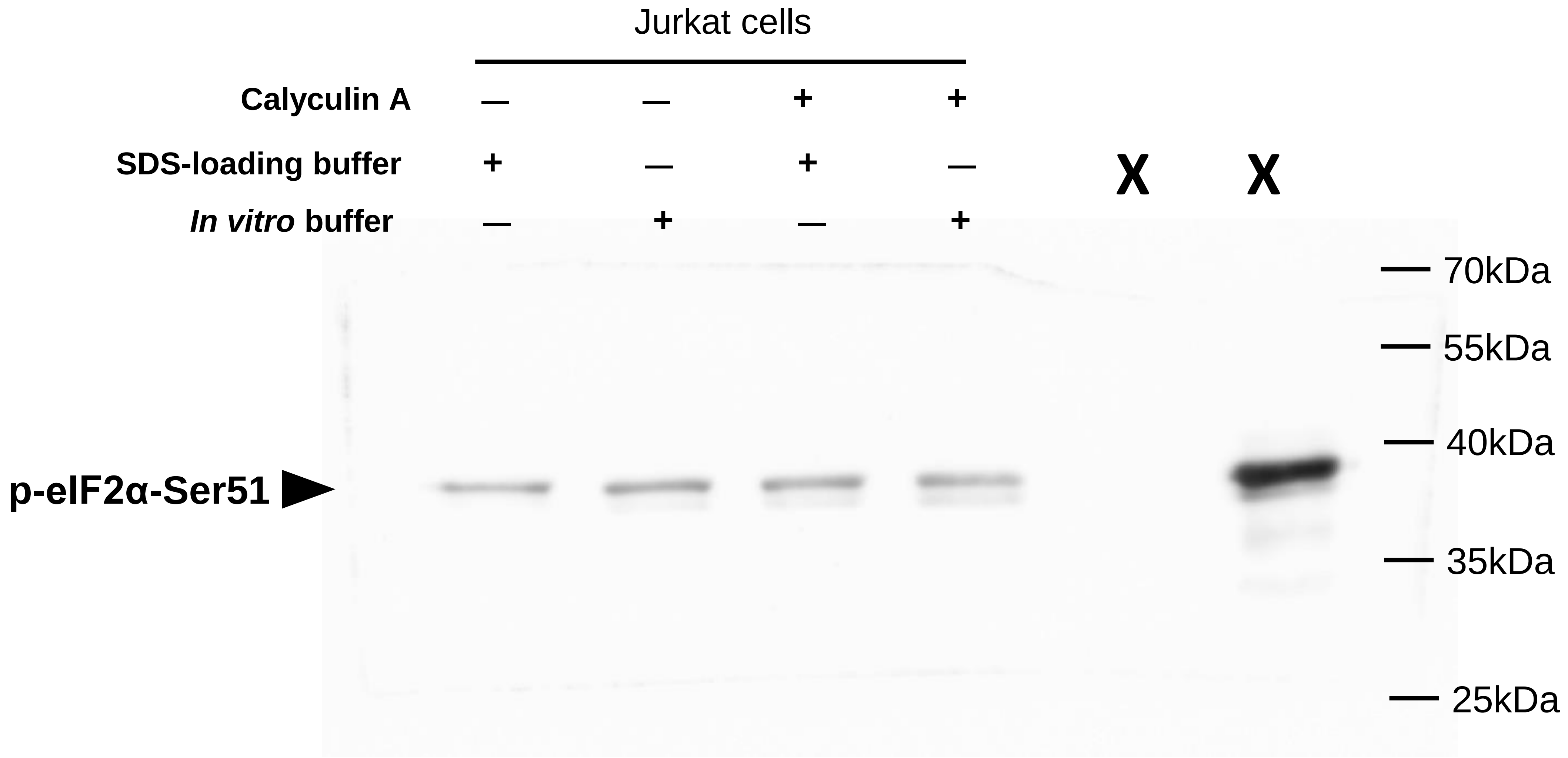

Fig. 1B
Upper panel

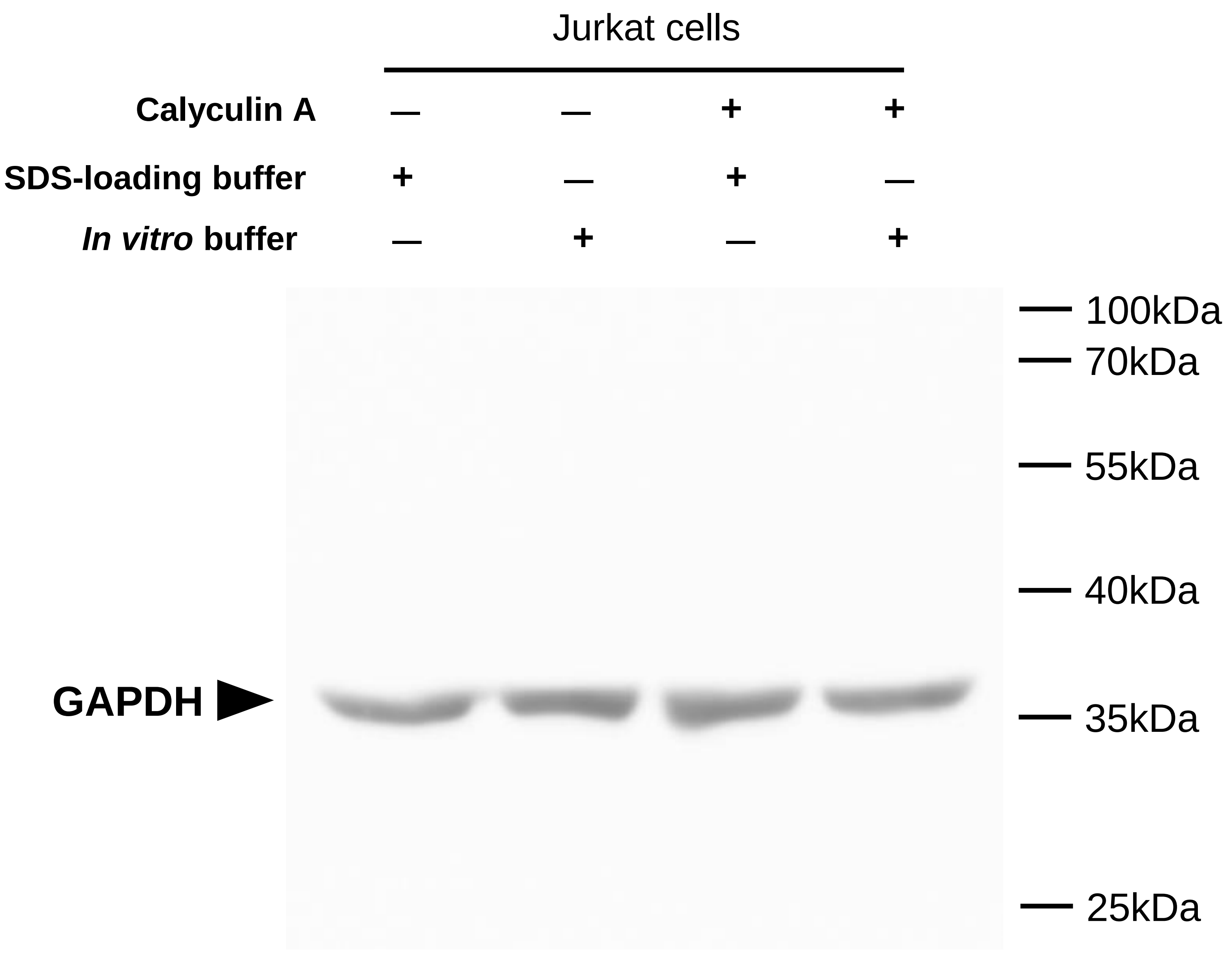

The proteins were detected using a Luminescent Image Analyzer (Fujifilm LAS-4000)

Fig. 1C

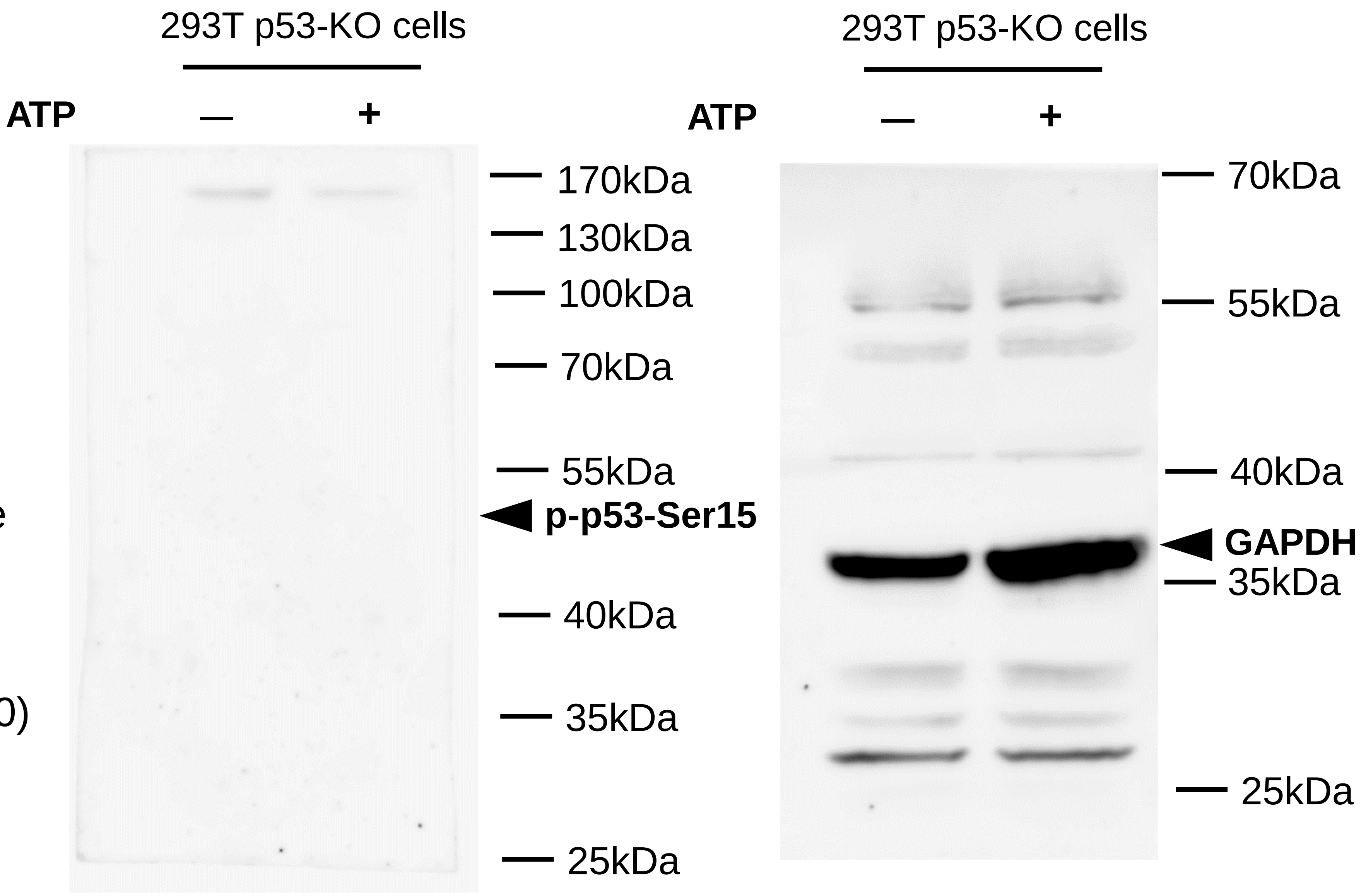

The proteins were detected using a Luminescent Image Analyzer (Fujifilm LAS-4000)

Fig. 1D

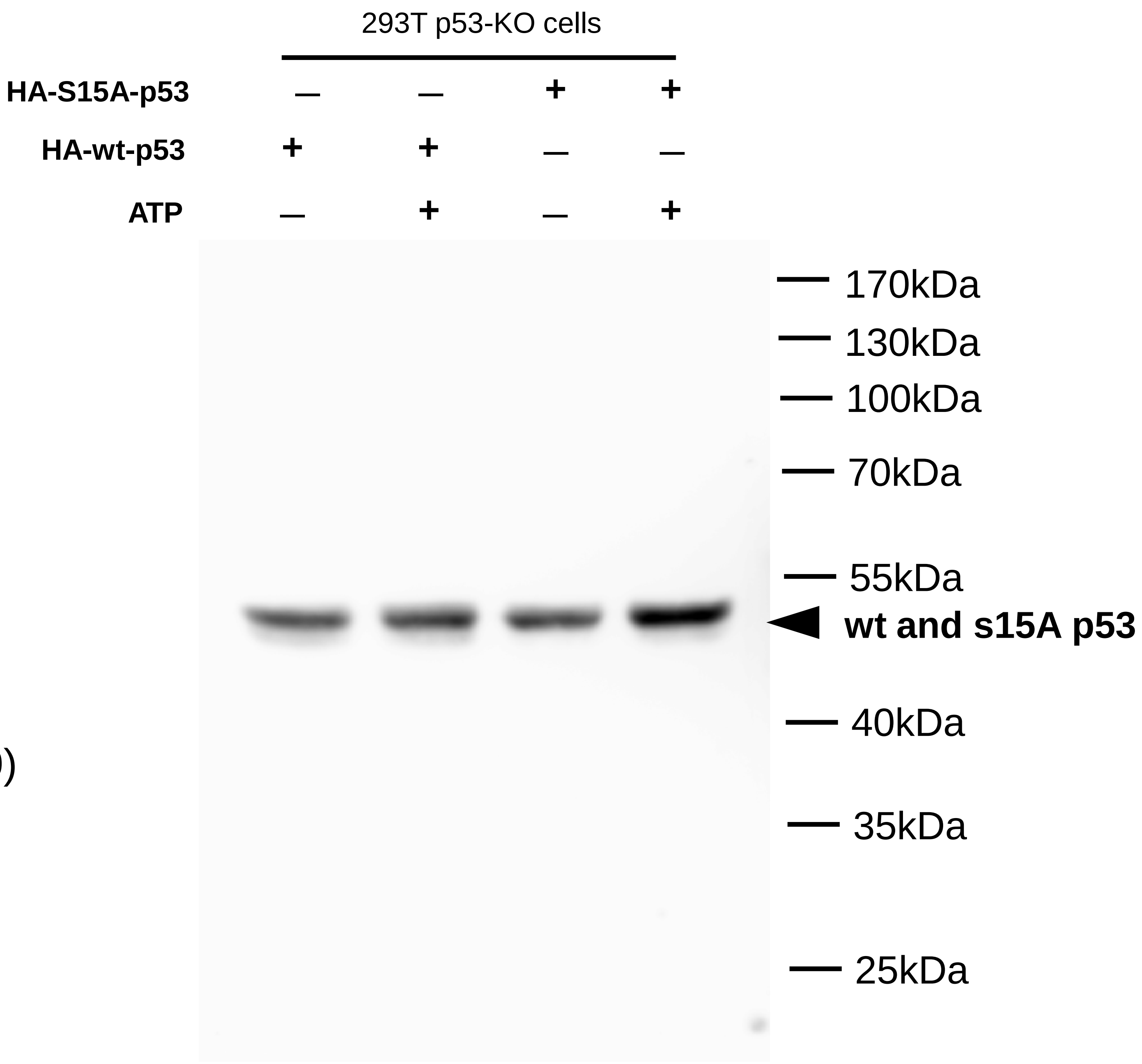

The proteins were detected using a Luminescent Image Analyzer (Fujifilm LAS-4000)

Fig. 1D

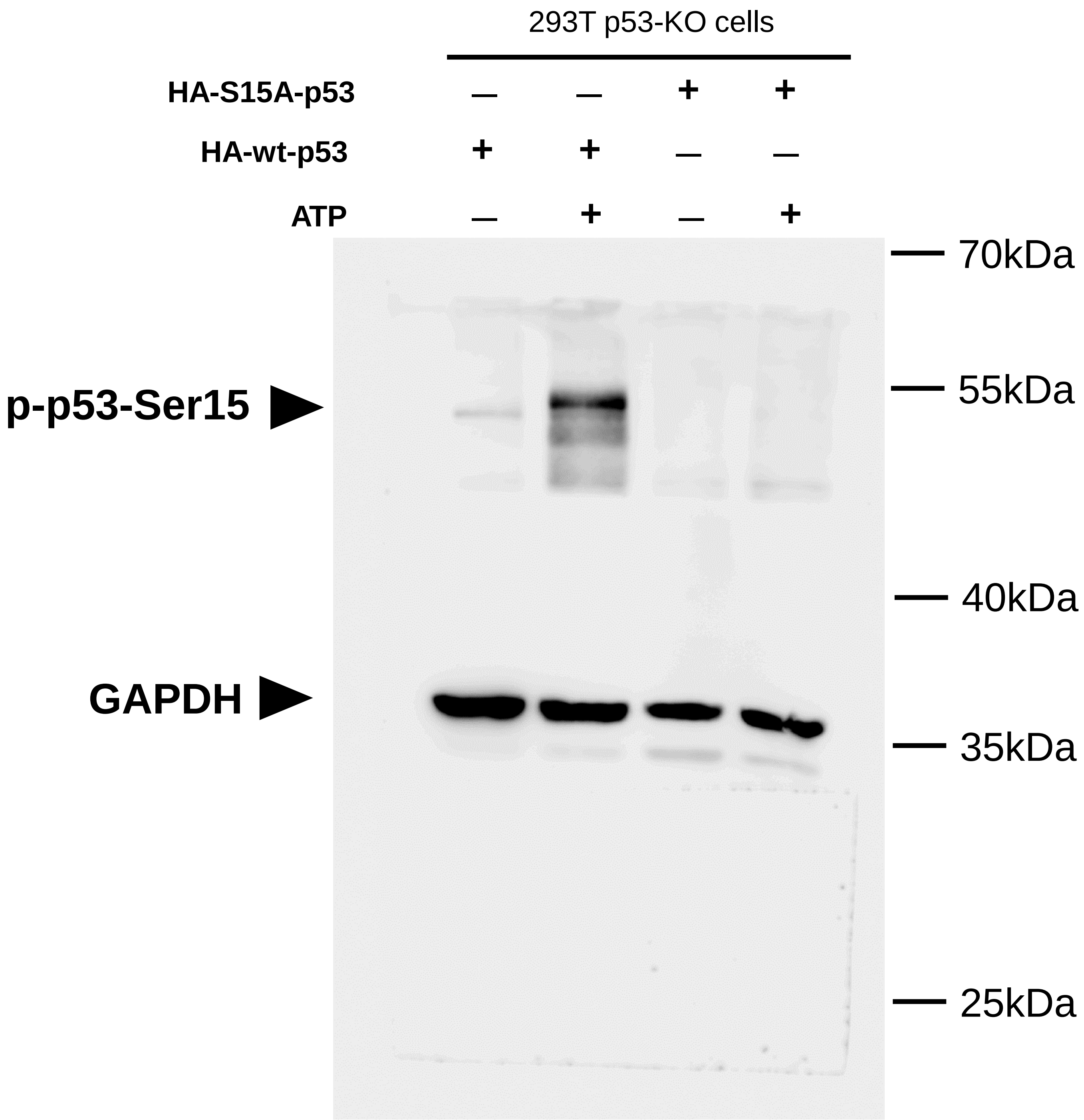

The proteins were detected using a Luminescent Image Analyzer (Fujifilm LAS-4000)

Fig. 1E

The proteins were detected using a Luminescent Image Analyzer (Fujifilm LAS-4000)

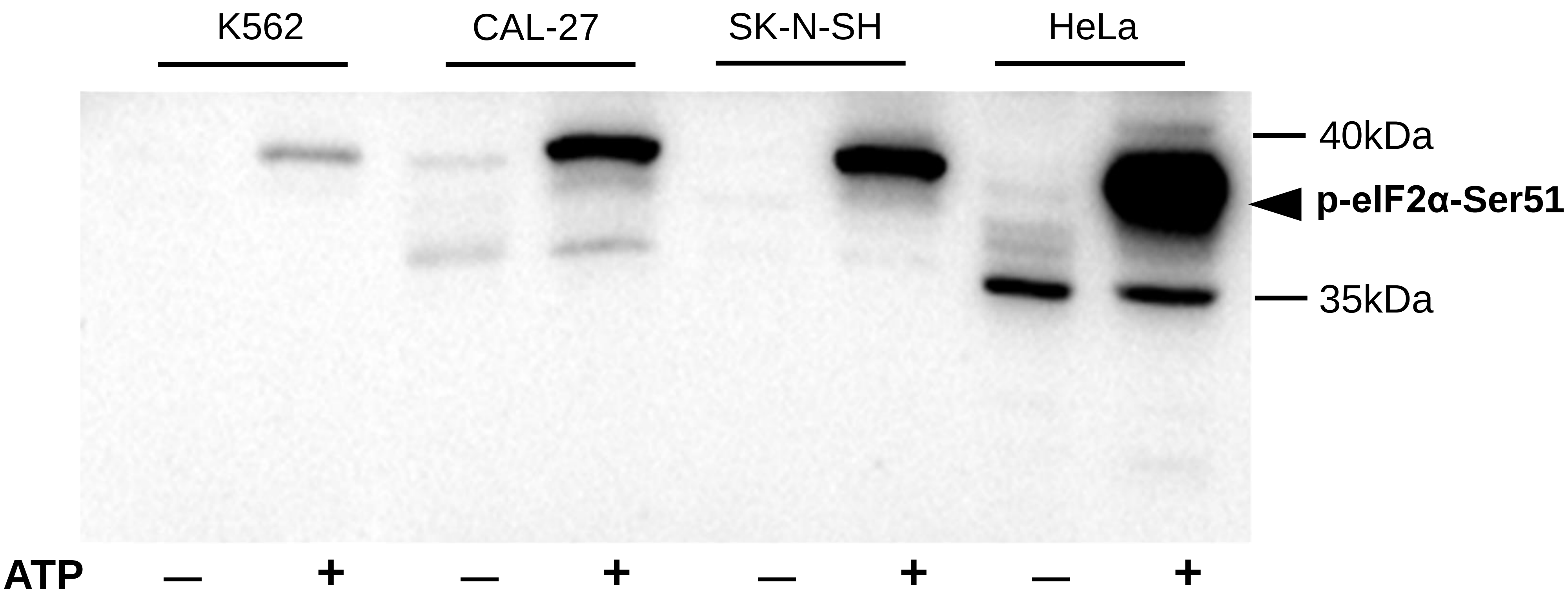

Fig. 1E

The proteins were detected using a Luminescent Image Analyzer (Fujifilm LAS-4000)

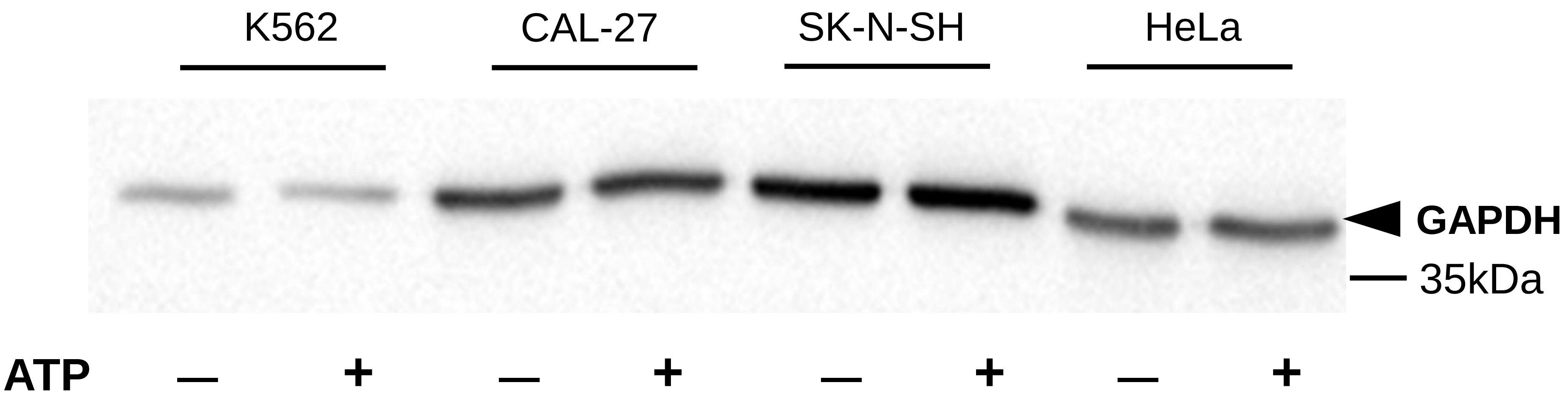

Fig. 1F

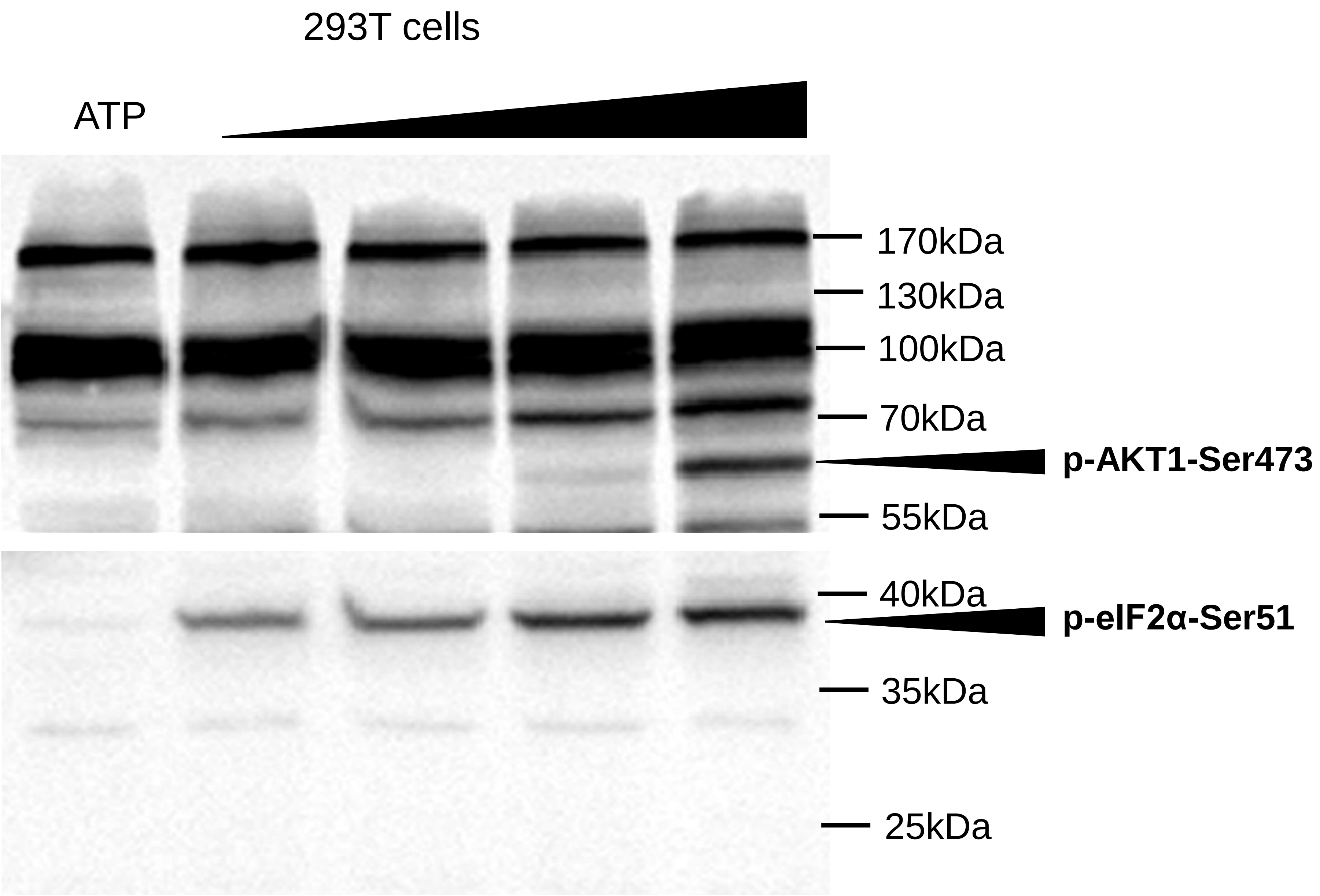

The proteins were detected using a Luminescent Image Analyzer (Fujifilm LAS-4000)

Fig. 1F

The proteins were detected using a Luminescent Image Analyzer (Fujifilm LAS-4000)

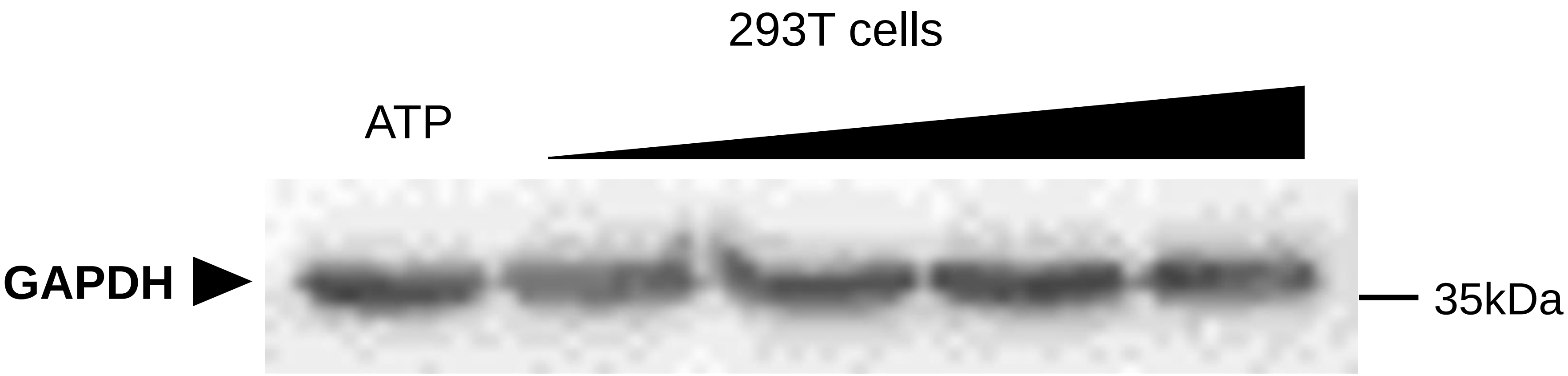

Fig. 2A

The proteins were detected using a Luminescent Image Analyzer (Fujifilm LAS-4000)

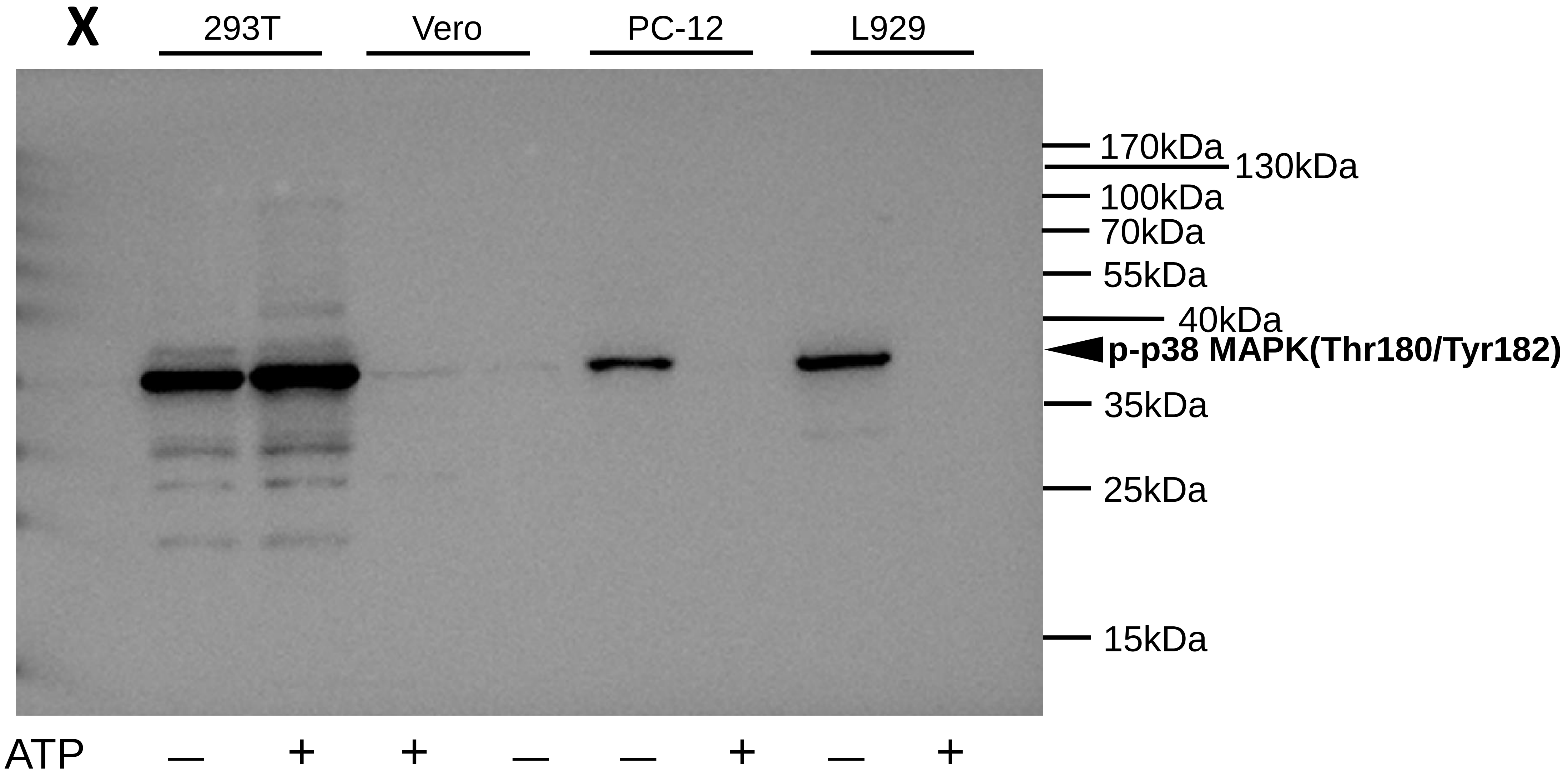

Fig. 2A

The proteins were detected using a Luminescent Image Analyzer (Fujifilm LAS-4000)

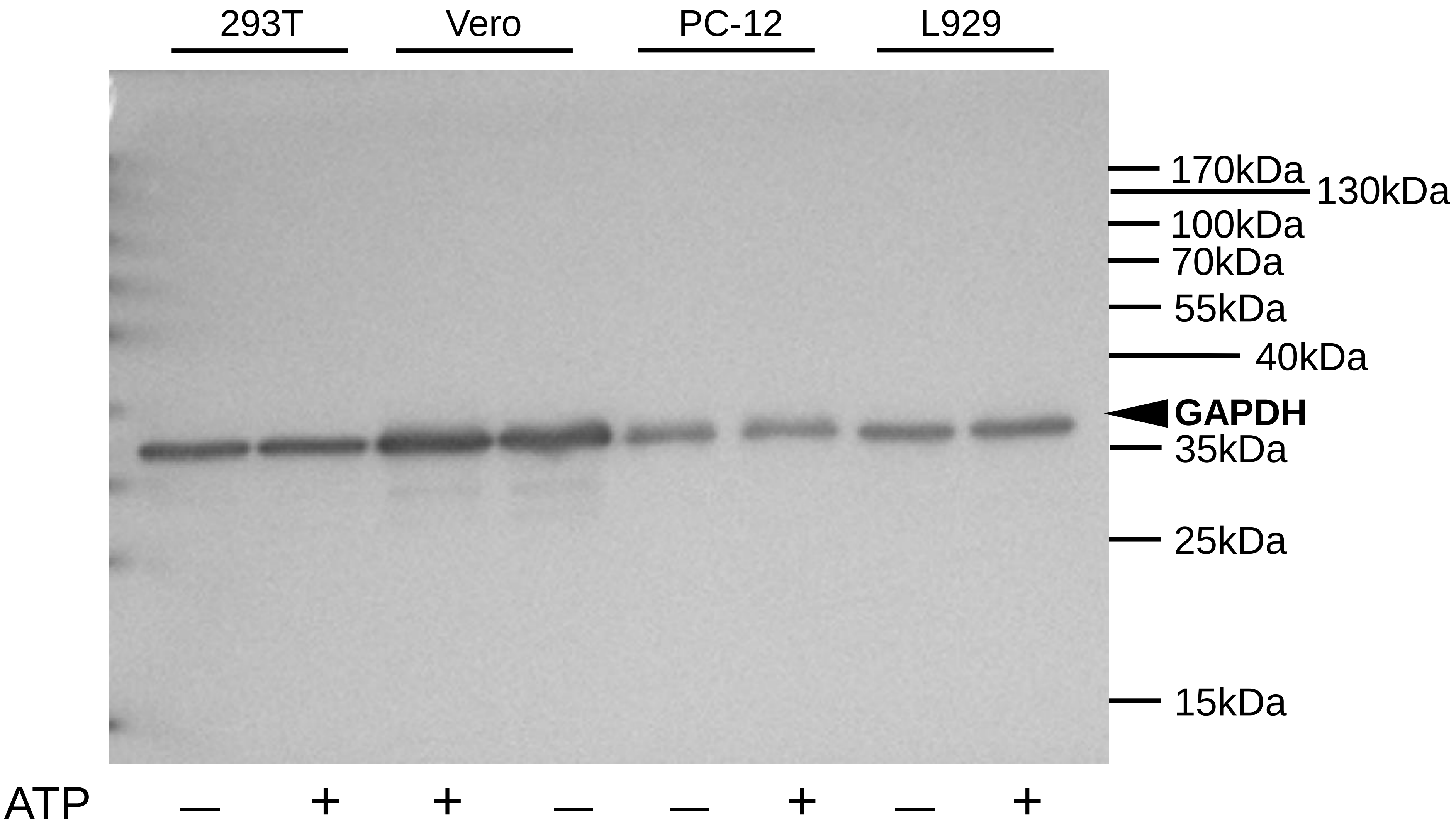

Fig. 2B

The proteins were detected using a Luminescent Image Analyzer (Fujifilm LAS-4000)

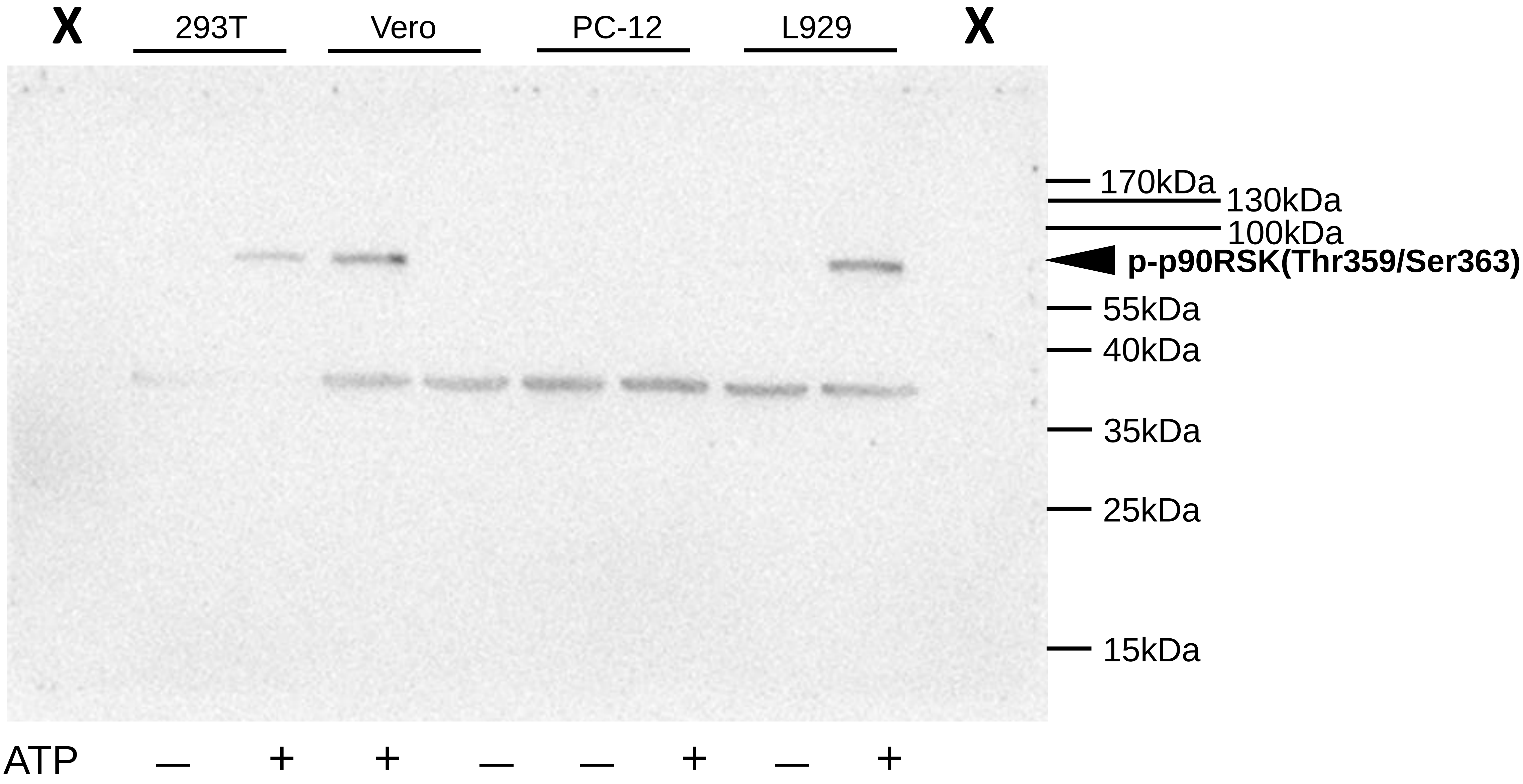

Fig. 2B

The proteins were detected using a Luminescent Image Analyzer (Fujifilm LAS-4000)

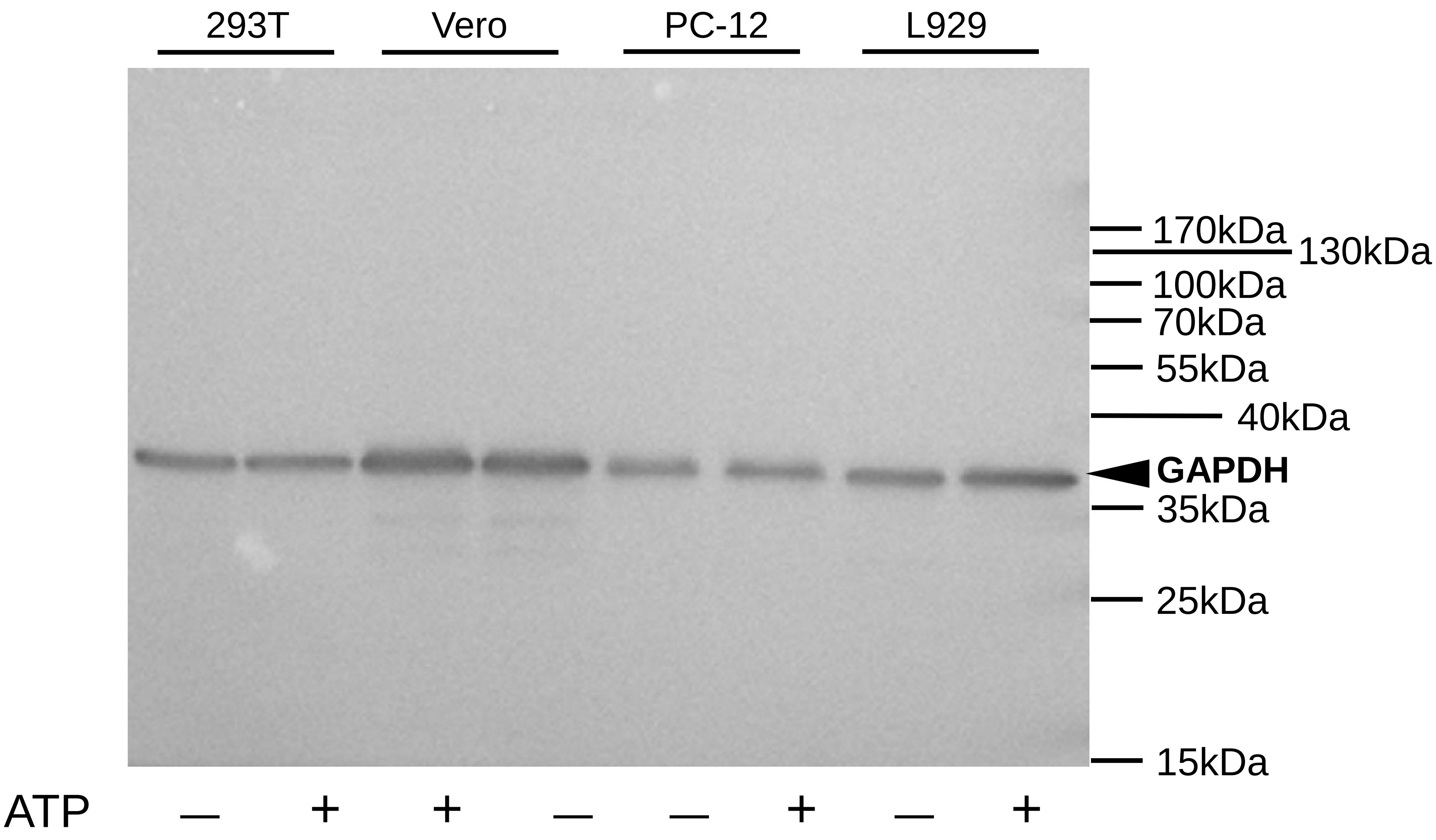

Fig. 2C

The proteins were detected using a Luminescent Image Analyzer (Fujifilm LAS-4000)

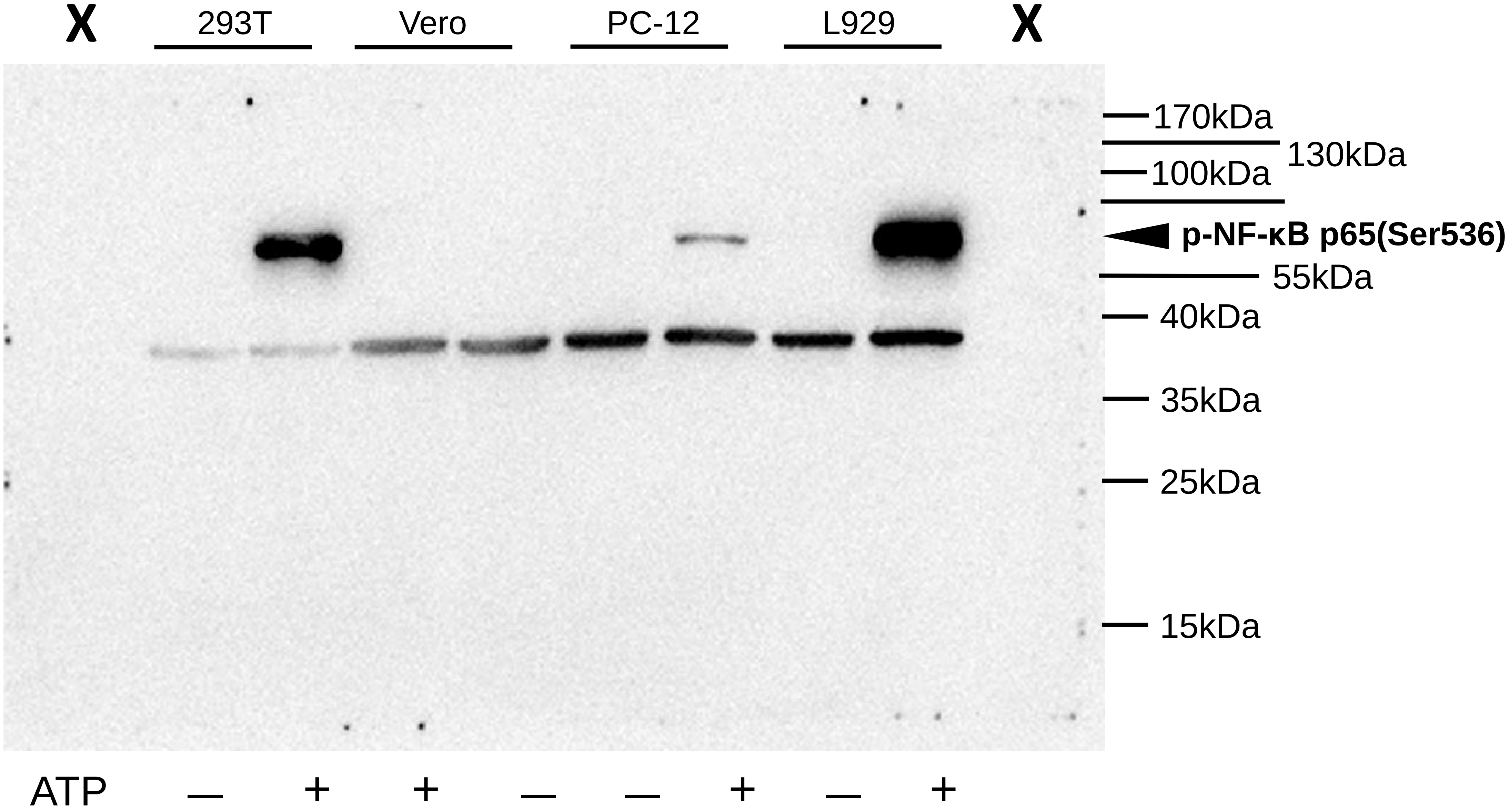

Fig. 2C

The proteins were detected using a Luminescent Image Analyzer (Fujifilm LAS-4000)

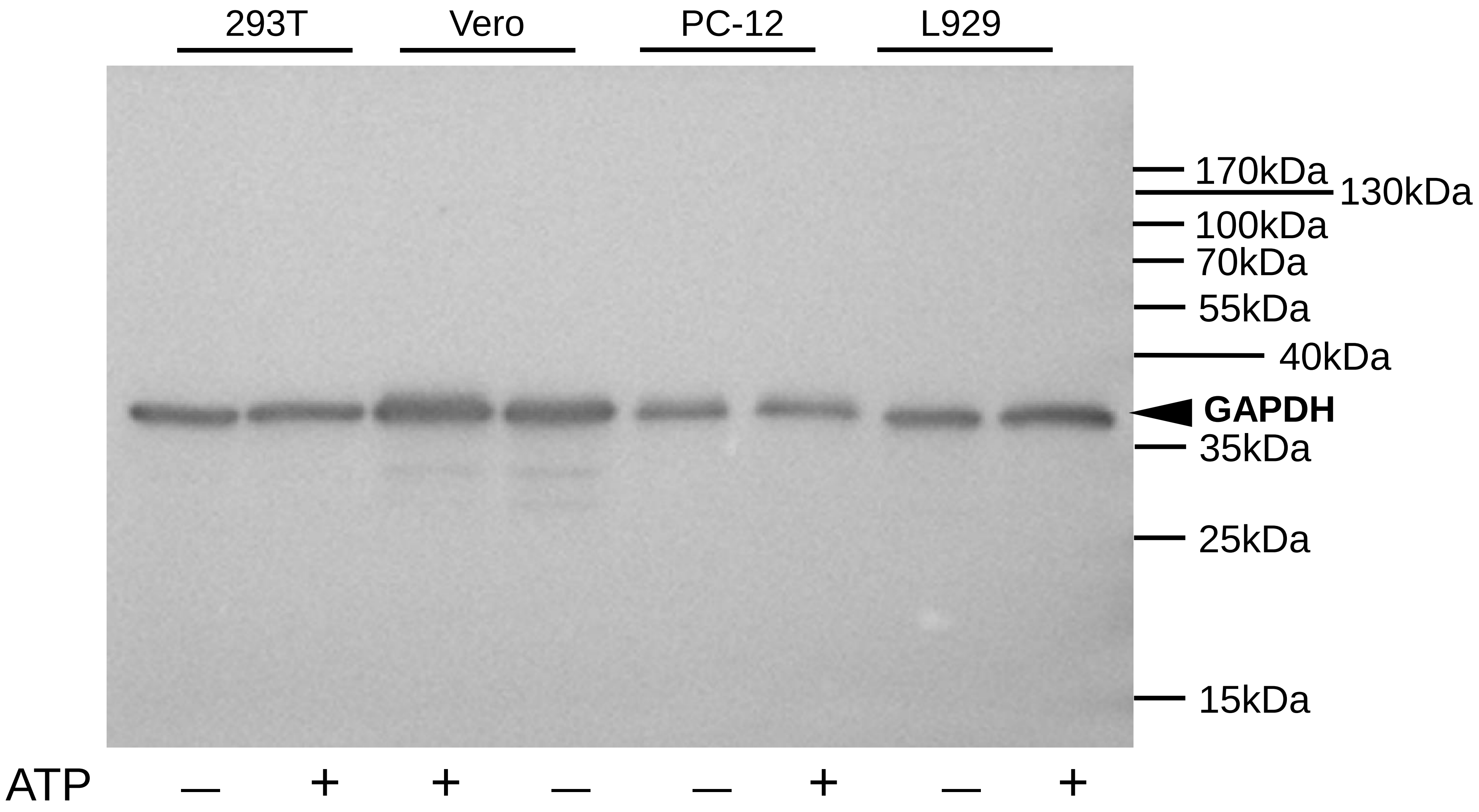

Fig. 2D
Left panel

The proteins were detected using a Luminescent Image Analyzer (Fujifilm LAS-4000)

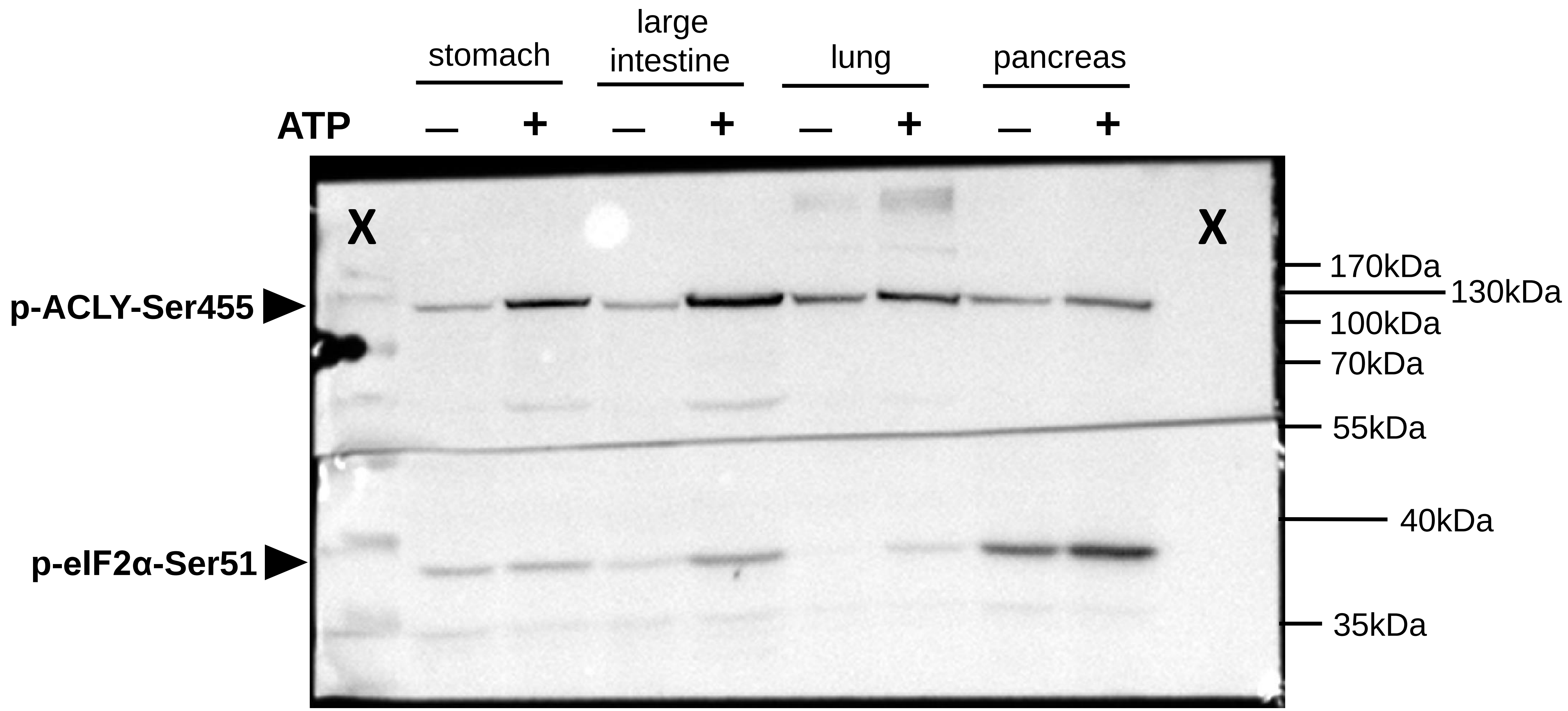

Fig. 2D
Right panel

The proteins were detected using a Luminescent Image Analyzer (Fujifilm LAS-4000)

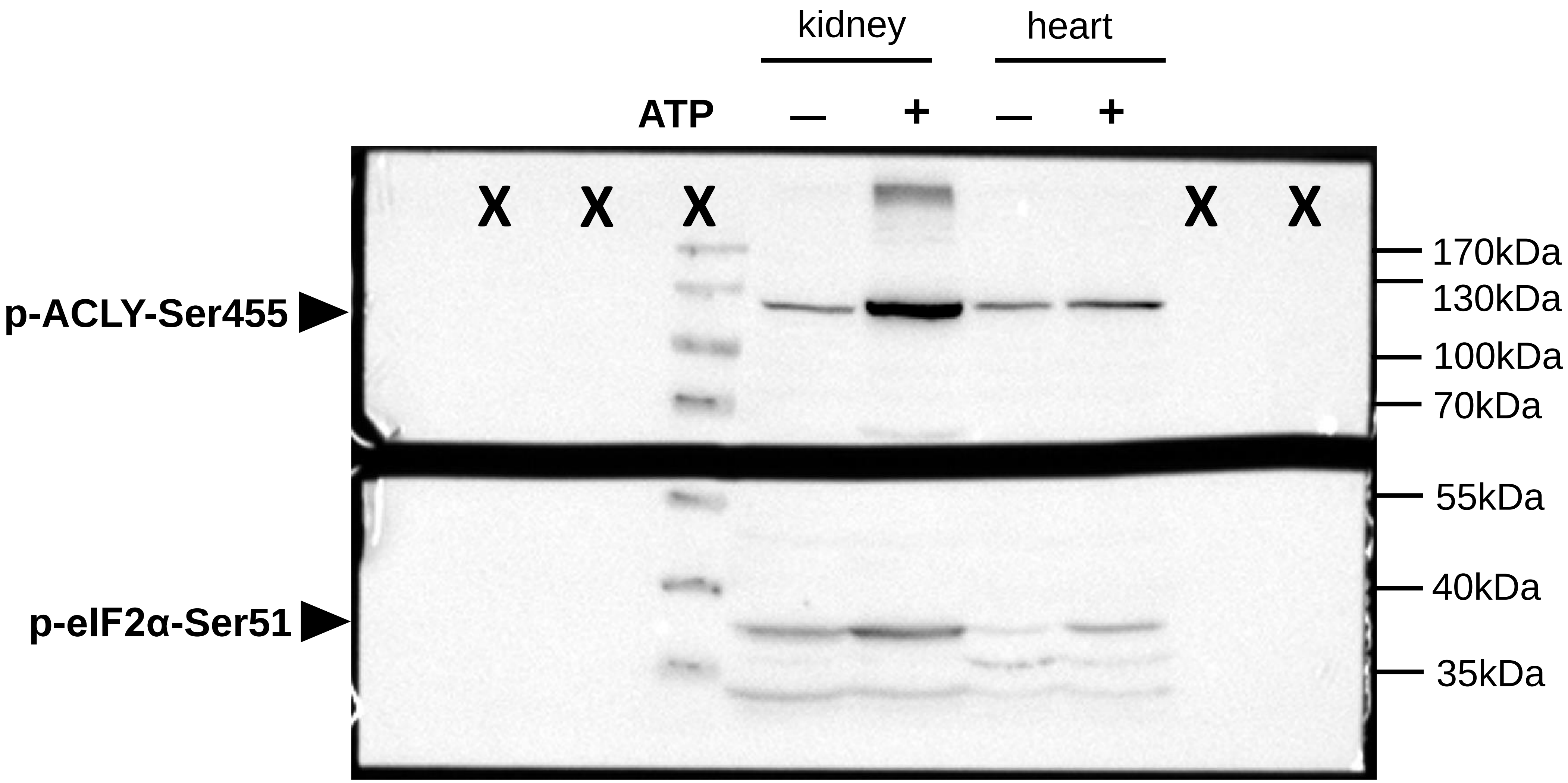

Fig. 2D
Left panel

The proteins were detected using a Luminescent Image Analyzer (Fujifilm LAS-4000)

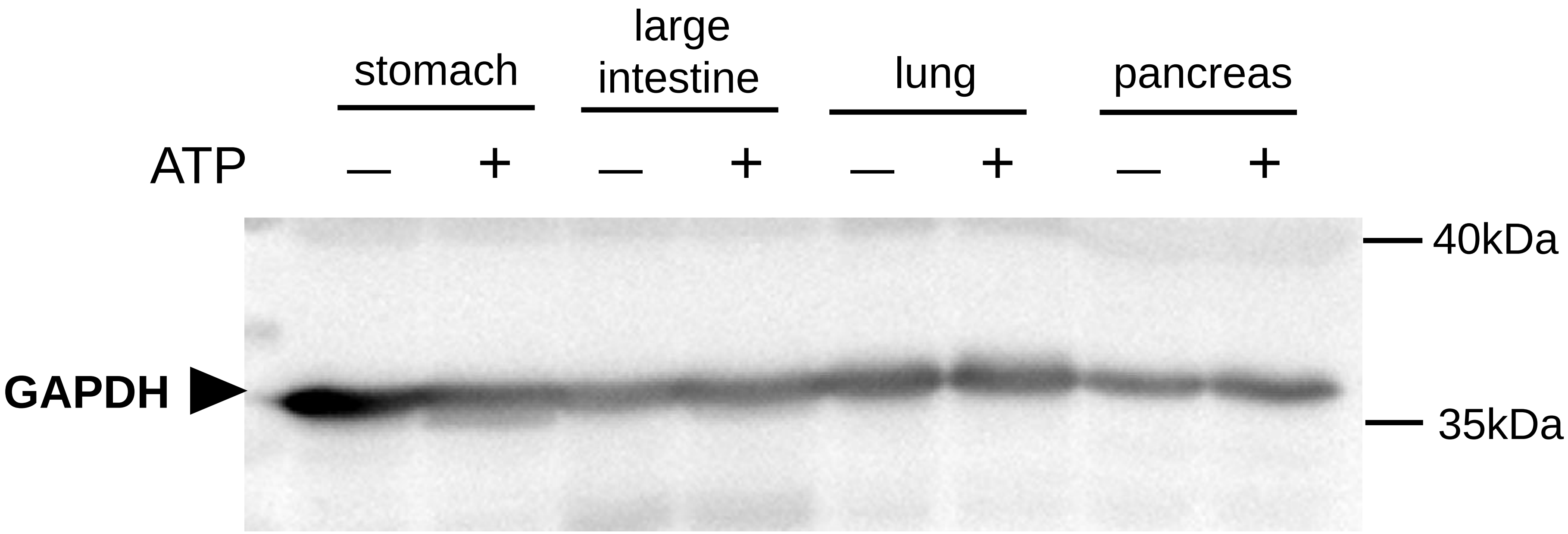

Fig. 2D
Right panel

The proteins were detected using a Luminescent Image Analyzer (Fujifilm LAS-4000)

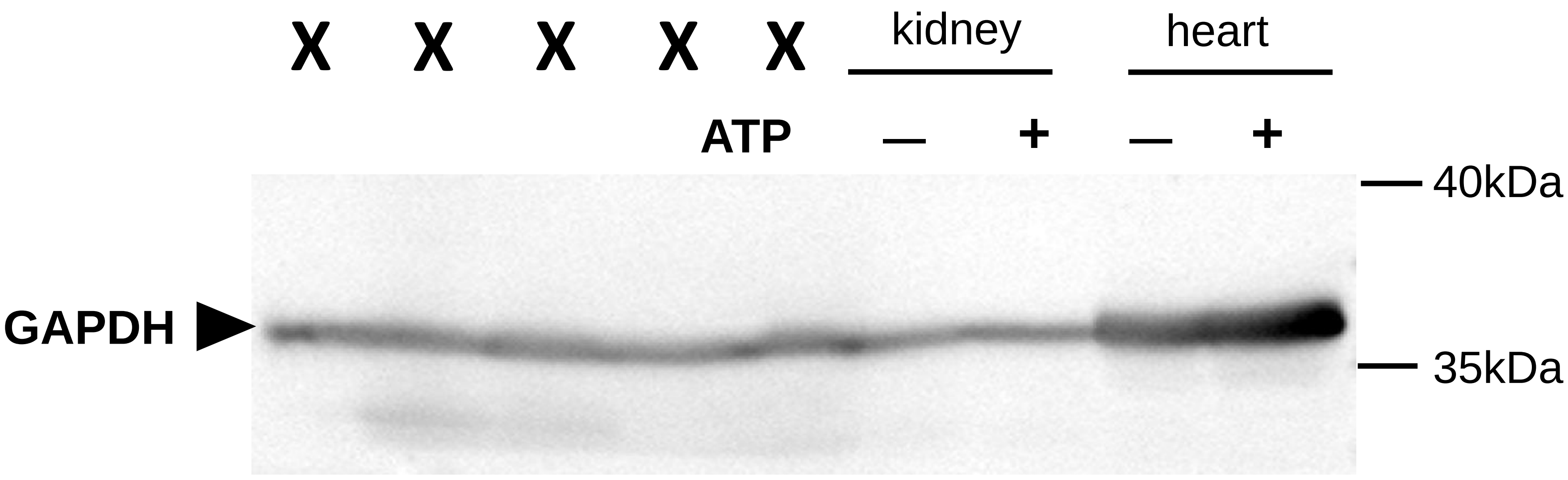

Fig. 3A

The proteins were detected using a Luminescent Image Analyzer (Fujifilm LAS-4000)

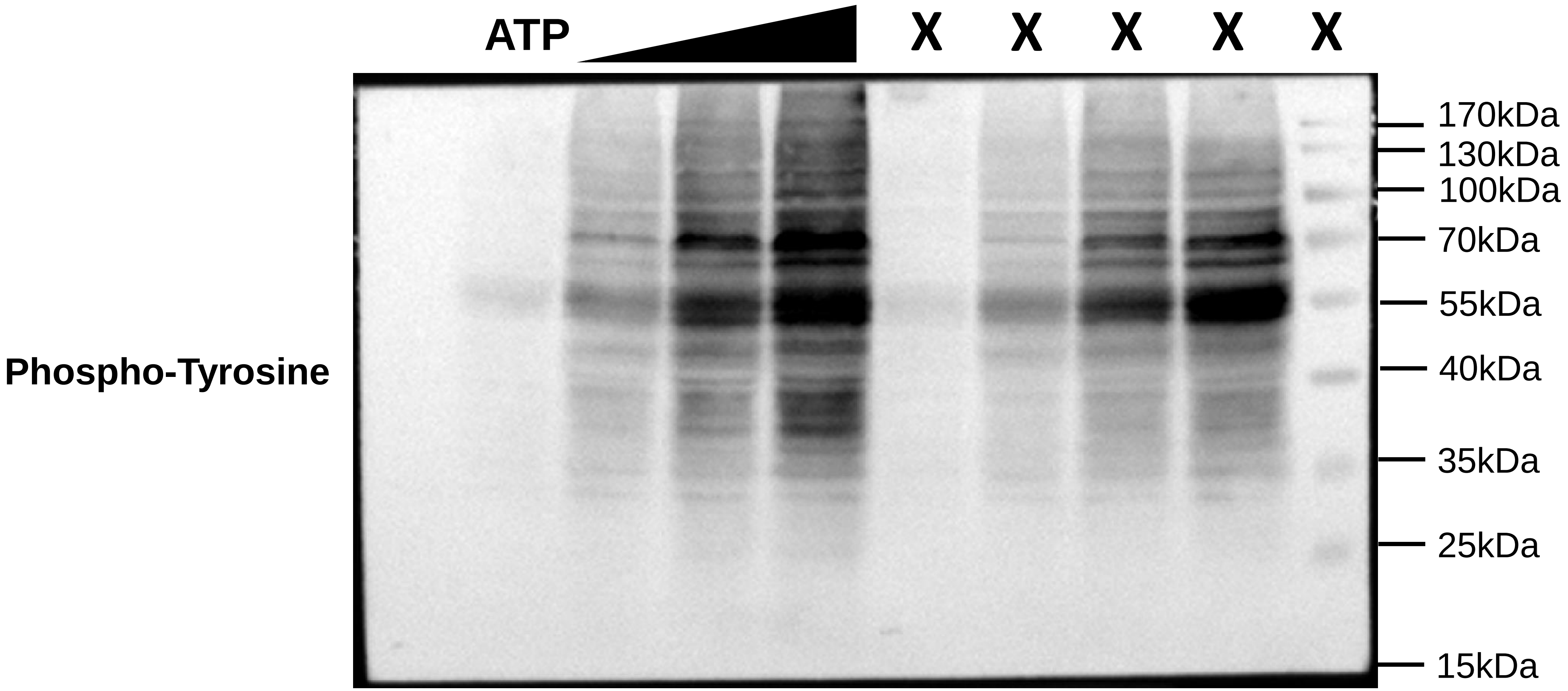

Fig. 3A

The proteins were detected using a Luminescent Image Analyzer (Fujifilm LAS-4000)

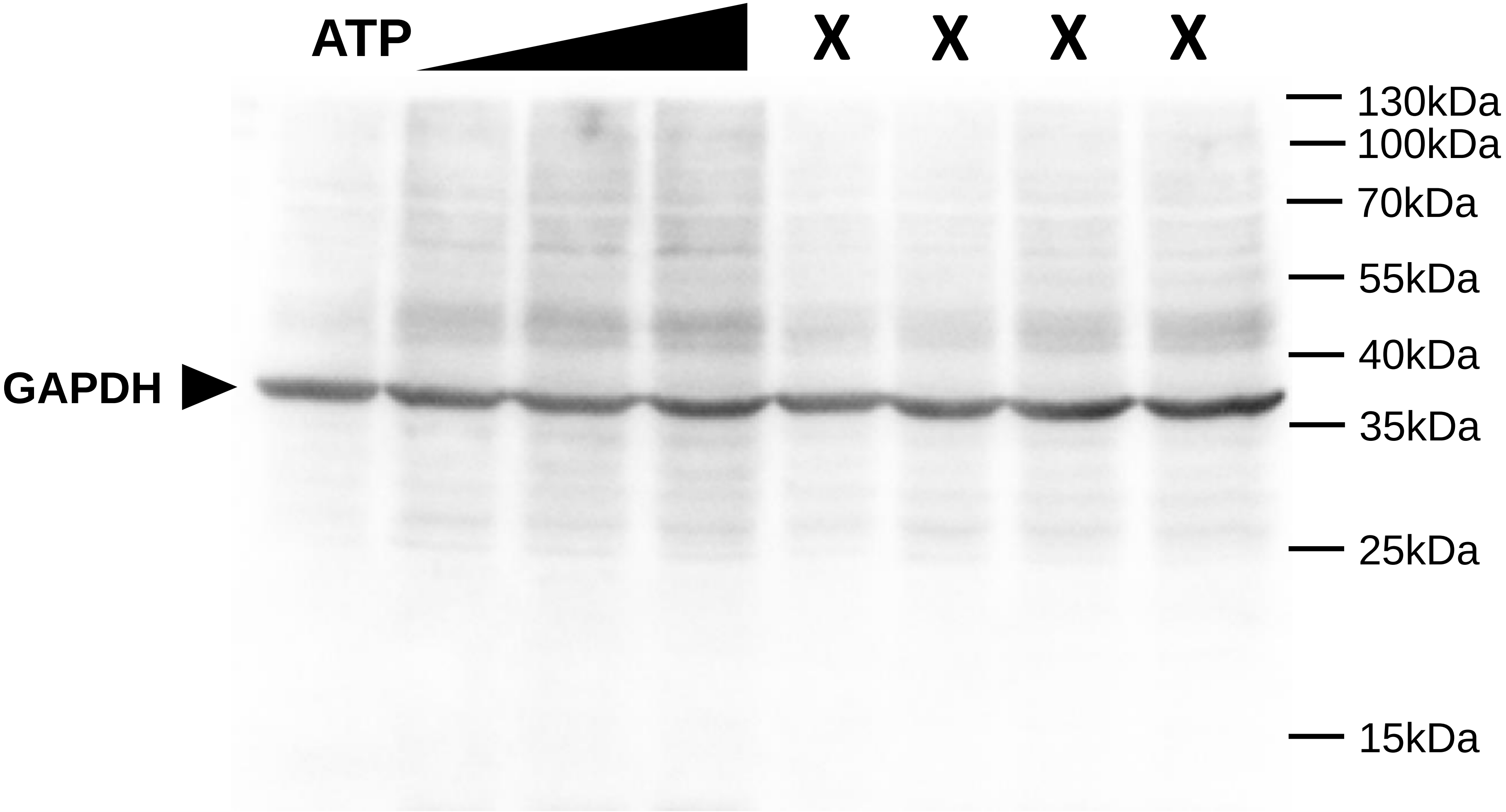

Fig. 3B

The proteins were detected using a Luminescent Image Analyzer (Fujifilm LAS-4000)

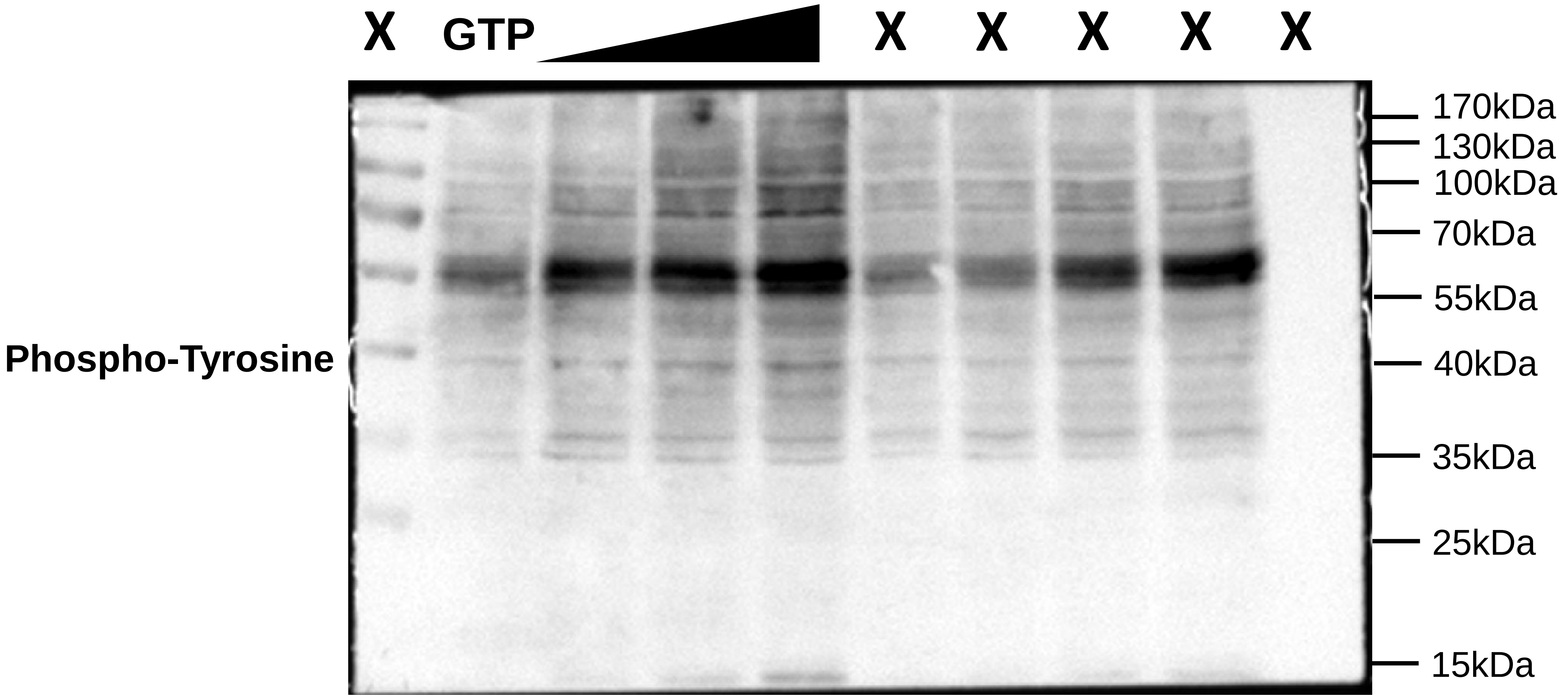

Fig. 3B

The proteins were detected using a Luminescent Image Analyzer (Fujifilm LAS-4000)

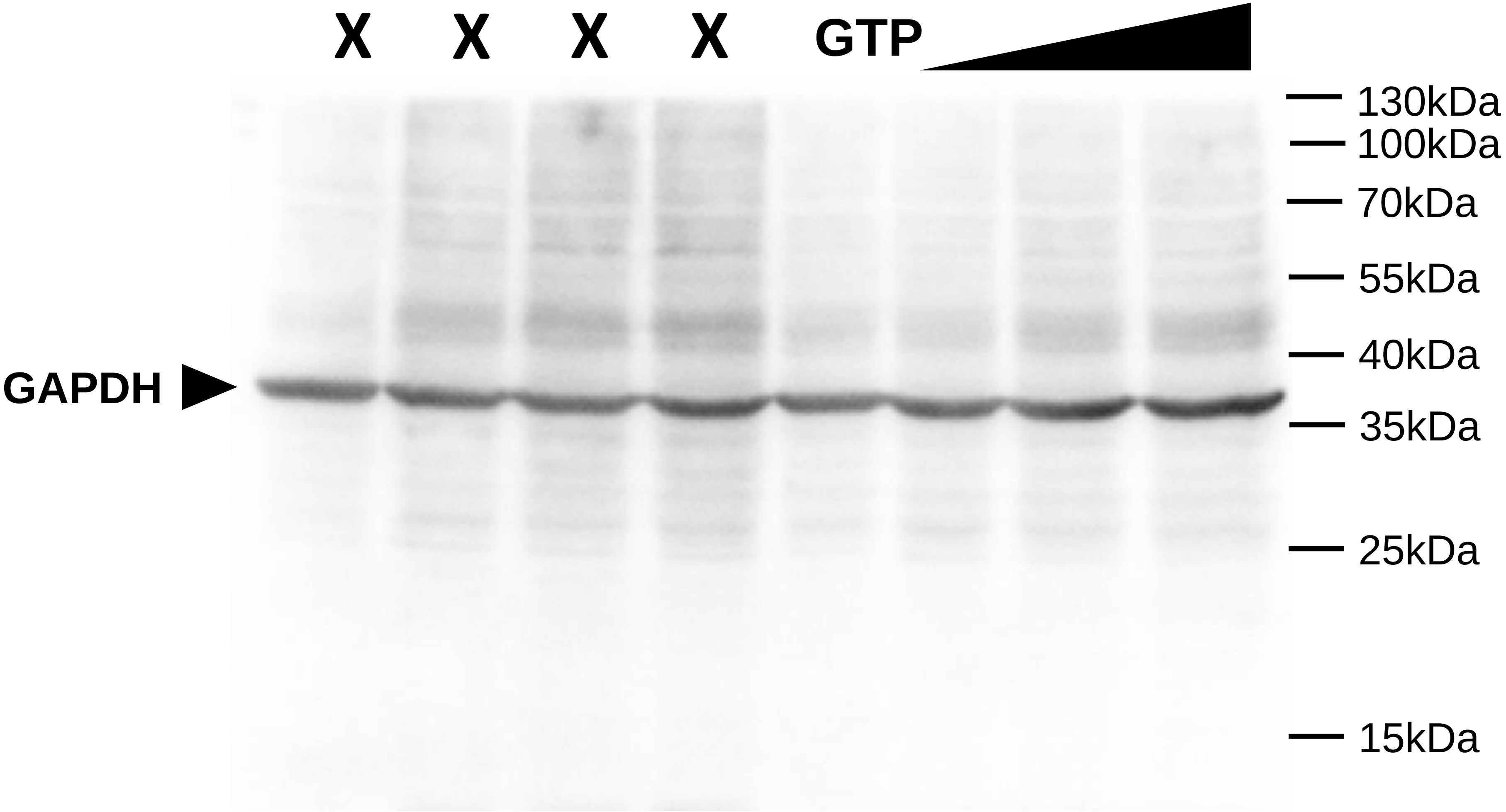

Fig. 3C

The proteins were detected using a Luminescent Image Analyzer (Fujifilm LAS-4000)

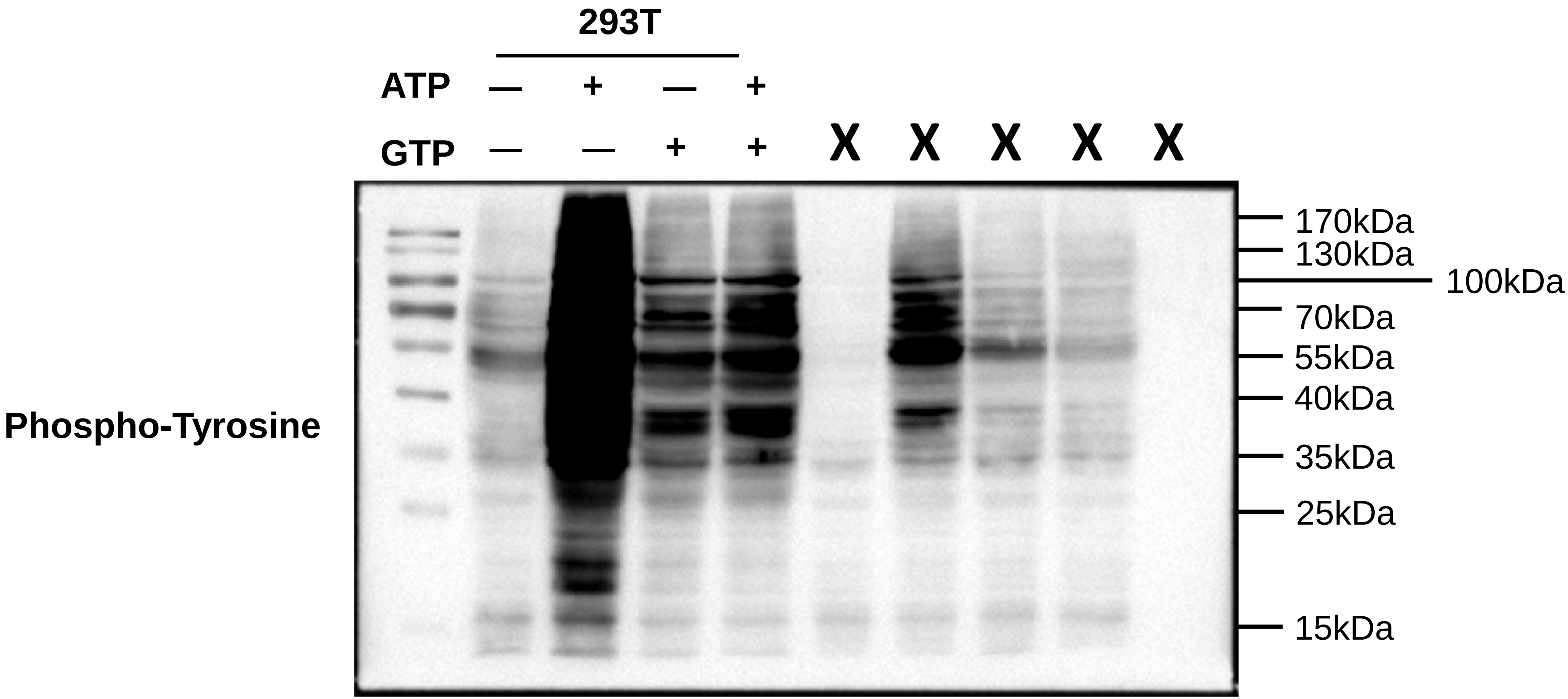

Fig. 3C

The proteins were detected using a Luminescent Image Analyzer (Fujifilm LAS-4000)

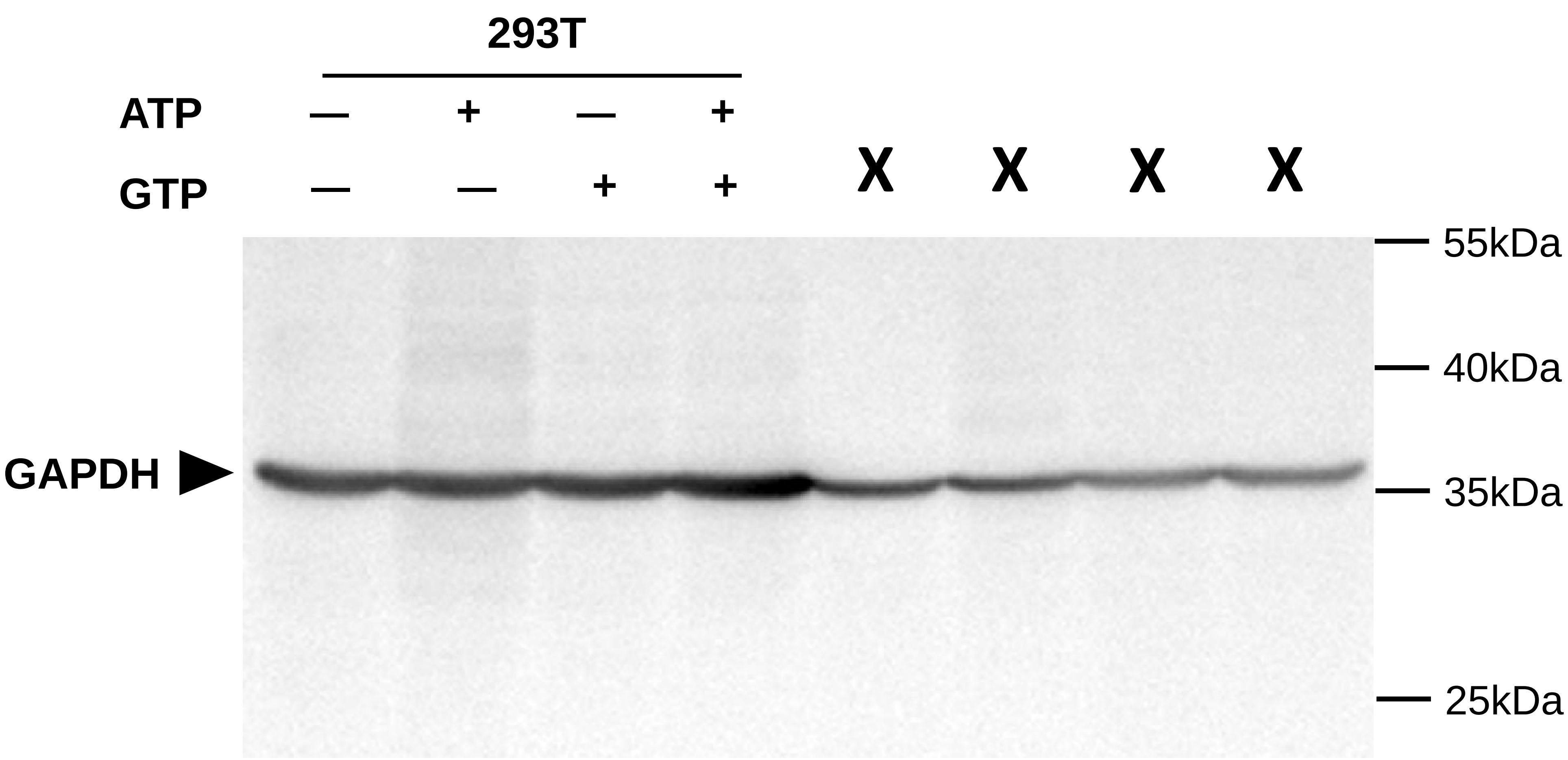

Fig. 3D

The proteins were detected using a Luminescent Image Analyzer (Fujifilm LAS-4000)

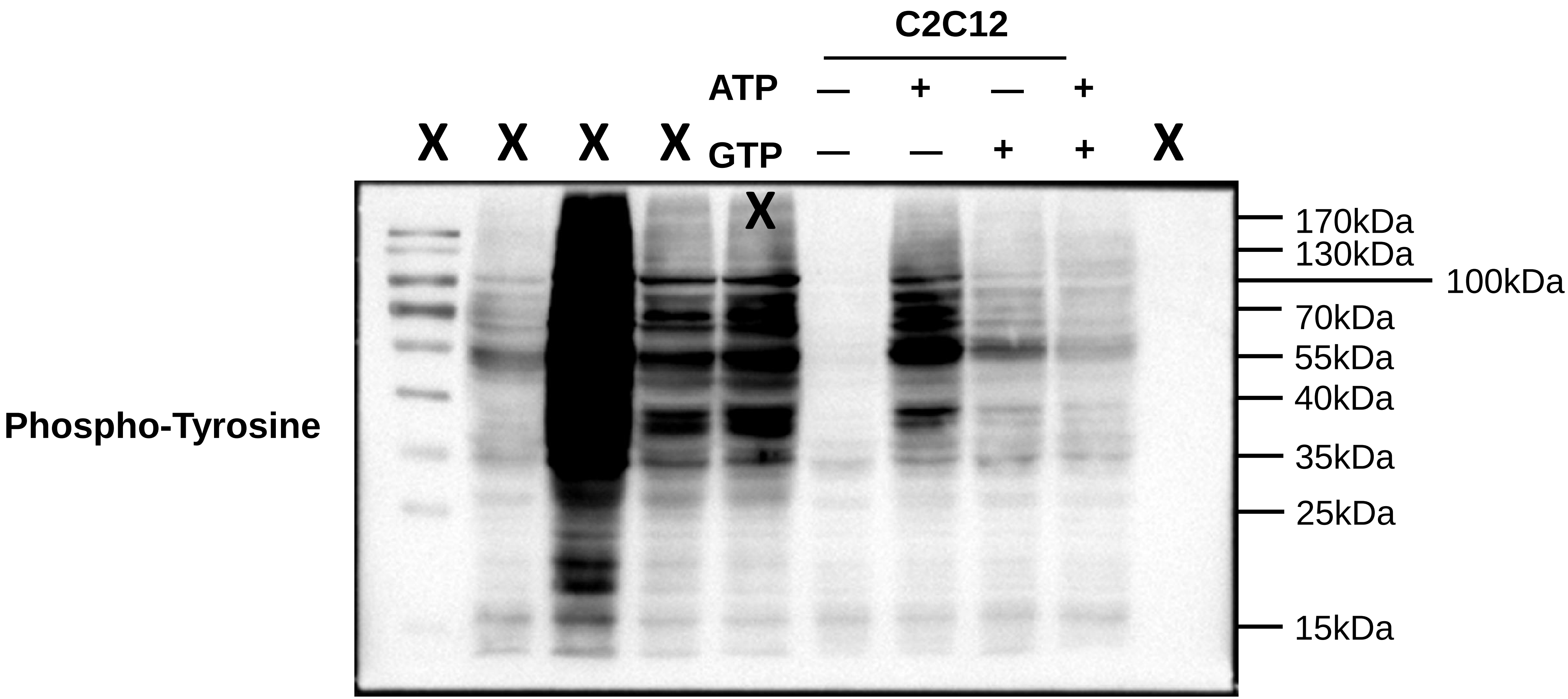

Fig. 3D

The proteins were detected using a Luminescent Image Analyzer (Fujifilm LAS-4000)

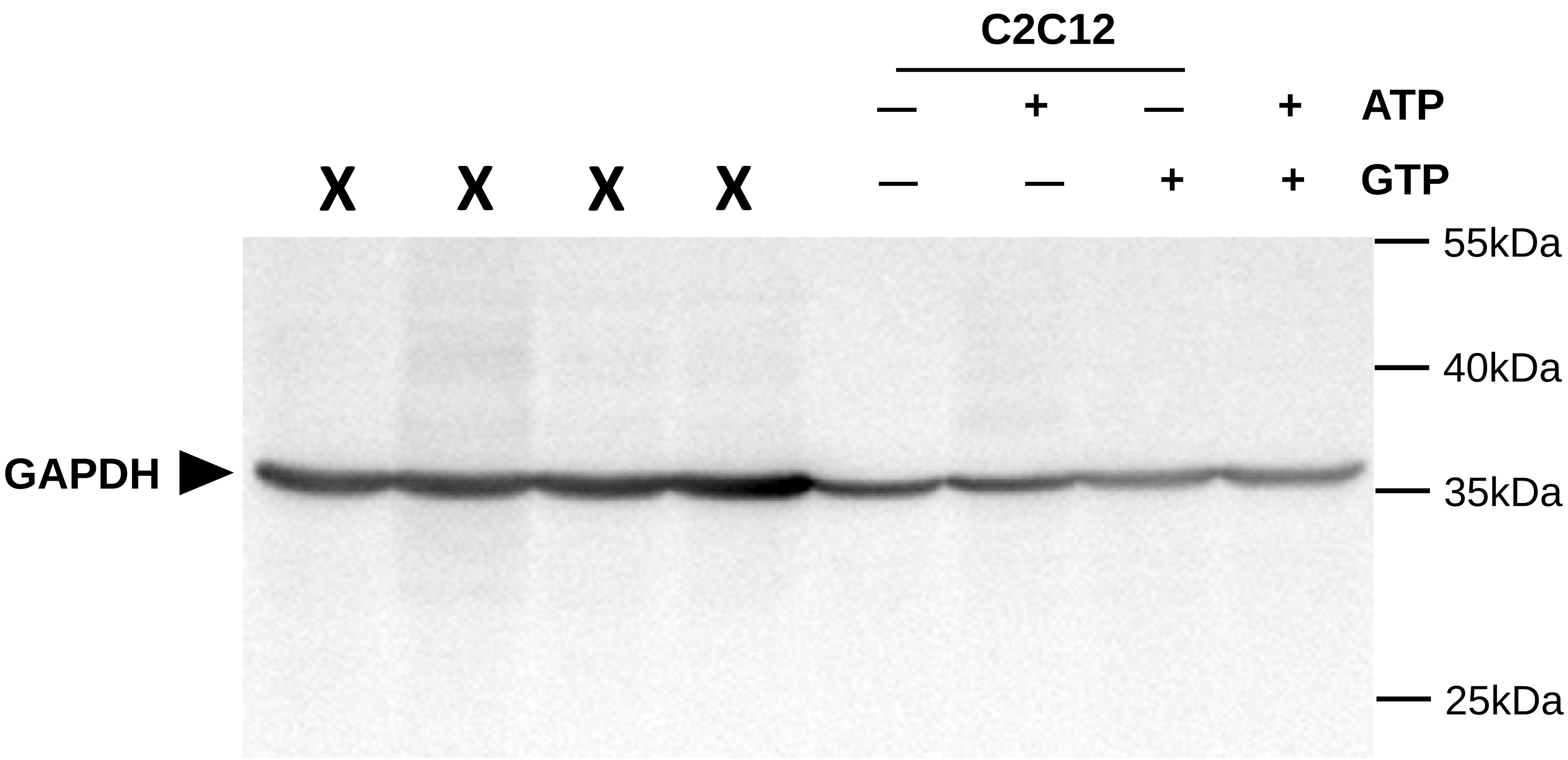

Fig. 3E

The proteins were detected using a Luminescent Image Analyzer (Fujifilm LAS-4000)

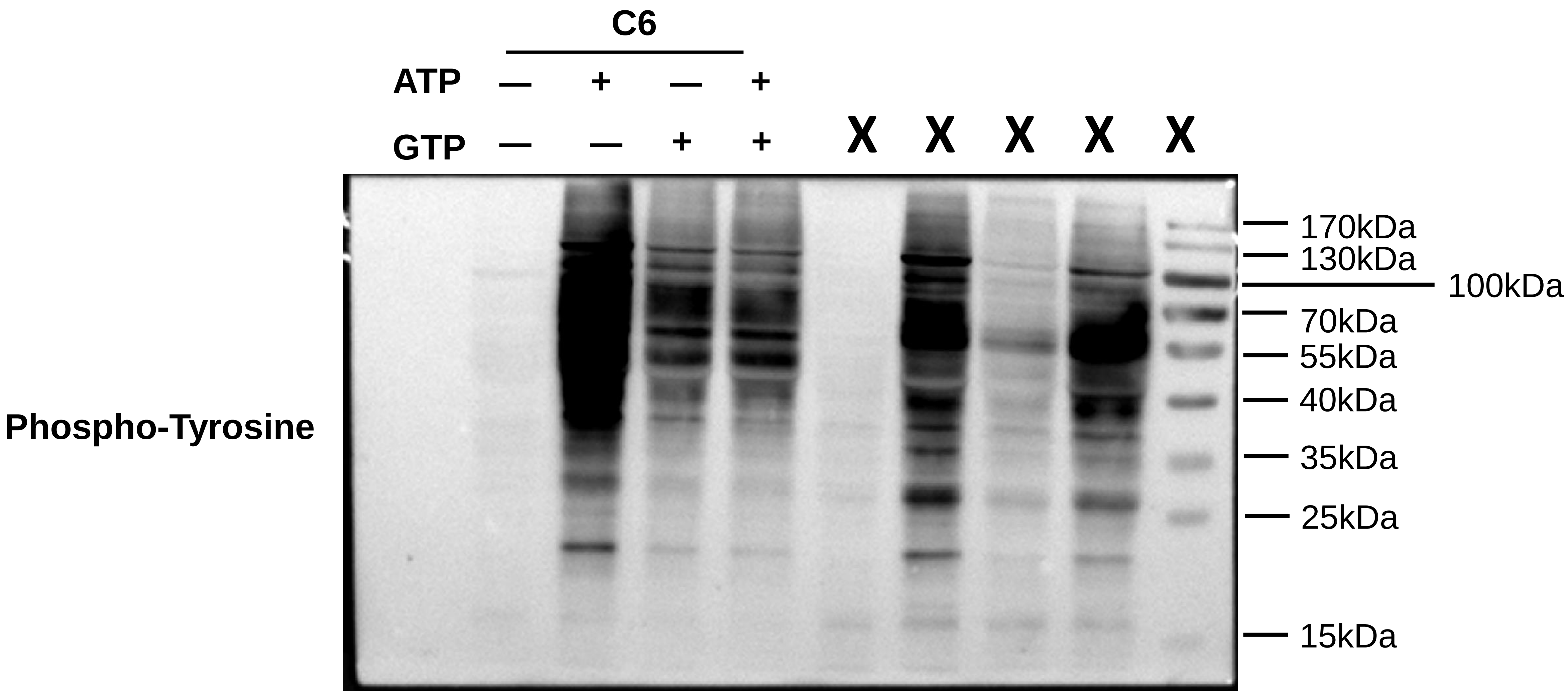

Fig. 3E

The proteins were detected using a Luminescent Image Analyzer (Fujifilm LAS-4000)

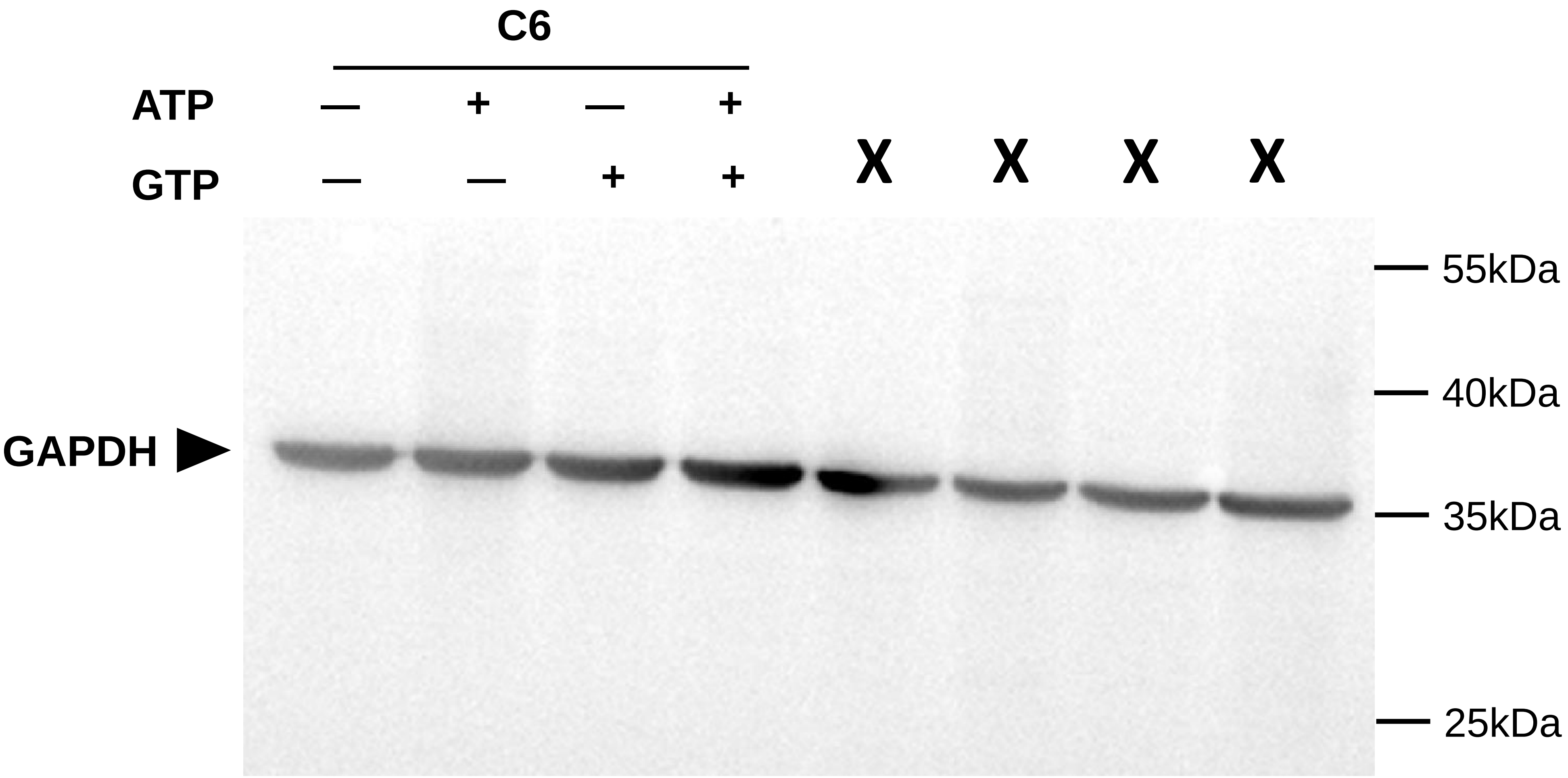

Supplement: S1 Raw images — (PDF) [file pone.0272138.s003.pdf]
